# Mendelian randomization study reveals a causal relationship between adiponectin and LDL cholesterol in Africans

**DOI:** 10.1038/s41598-022-21922-w

**Published:** 2022-11-08

**Authors:** Karlijn A. C. Meeks, Amy R. Bentley, Ayo P. Doumatey, Adebowale A. Adeyemo, Charles N. Rotimi

**Affiliations:** Center for Research on Genomics and Global Health, National Human Genome Research Institute, National Institutes of Health, 12 South Drive Bldg 12A ste 1025, Bethesda, MD 20892-5611 USA

**Keywords:** Population genetics, Predictive markers, Cardiovascular biology

## Abstract

Adiponectin has been associated with cardiometabolic traits in observational studies across populations, yet it is unclear if these associations are causal. We performed Mendelian randomization (MR) analysis to assess the relationship between adiponectin and cardiometabolic traits in sub-Saharan Africans. We constructed a polygenic risk score (PRS) for adiponectin levels across 3354 unrelated sub-Saharan Africans. The PRS was used as the instrumental variable in two-stage least-squares MR analysis to assess its association with insulin resistance, HDL, LDL, total cholesterol, triglycerides, blood pressure, Type 2 Diabetes (T2D), and hypertension. The adiponectin PRS was causally related with LDL (β = 0.55, 95%CI 0.07–1.04, *P-*value = 0.024) but not the other traits. This association was observed in both overweight/obese and normal weight individuals, but only reached statistical significance among overweight/obese individuals (β = 0.55, 95%CI 0.01–1.08, *P*-value = 0.045). In normal weight individuals, the adiponectin PRS was associated with T2D (OR = 0.13, 95%CI 0.02–0.73, *P*-value = 0.021), and in men with HDL (β = 1.03, 95%CI 0.14–1.92, *P-value* = 0.023). The findings of this first MR study in sub-Saharan Africans support a causal relationship of adiponectin with LDL, with T2D in normal weight individuals only, and with HDL in men only. These observations add to the small but growing literature on adiponectin MR studies.

## Introduction

Nearly 80% of cardiovascular disease (CVD) related deaths occur in low- and middle-income countries^1^. In sub-Saharan Africa, the rates of CVD risk factors, such as type 2 diabetes (T2D) and hypertension, are increasing rapidly. Sub-Saharan Africa has the highest projected increase in T2D of all world regions with a projected increase of 156% by 2045^2^. The prevalence of hypertension in Africa has increased from 19.7% in 1990 to 30.8% in 2010^3^. A rise in obesity is thought to underly the increases in these cardiometabolic outcomes. Due to epidemiological transition, including reduced levels of physical activity and increased consumption of unhealthy diets, the obesity prevalence in sub-Saharan Africa has increased from 3 to 11% between 1975 and 2014^4^. However, a high prevalence of T2D has also been observed among normal weight Africans (Body Mass Index [BMI] < 25 kg/m^2^) living in Africa and in Europe^5,6^, for reasons that are not yet fully understood.

The biological mechanisms underlying the association of adiposity with cardiometabolic outcomes have been under intense study, especially with regard to the role of inflammation and of biomolecules secreted by adipose tissue, adipokines. Adipose tissue is an active organ involved in the production of a wide range of circulating proteins including adiponectin^7^. Adiponectin is an anti-inflammatory adipokine, which regulates glucose levels and fatty acid metabolism^8^. Higher circulating adiponectin levels have been negatively associated with obesity^9^, insulin resistance^9^, triglycerides^9^, and T2D risk^10^, and positively with high-density lipoprotein (HDL) cholesterol, and total cholesterol^9^ in epidemiological studies across populations, including sub-Saharan African populations^9^. However, these epidemiological studies are not able to determine whether changes in adiponectin levels are a cause or consequence of these cardiometabolic disorders. If the observed associations are causal and higher levels of adiponectin are indeed protective of cardiometabolic outcomes, there may be important therapeutic implications.

Mendelian randomization (MR) is a means of investigating causality between two traits. In MR analyses, the exposure is defined by genetic variants that contribute to the distribution of the putative causal agent. The random allocation of genetic variants is at the core of this technique as these variants can be used as proxies for the exposure that are not affected by confounding or reverse causation. MR studies assessing the causality of the association between adiponectin and cardiometabolic outcomes have generated contradictory results. Some found evidence for a causal effect on insulin resistance^11–13^ and T2D^11^, while findings of others did not support a causal relationship with either insulin resistance or T2D^14,15^. Similarly, causal associations were found with triglycerides and HDL cholesterol, but not with low-density lipoprotein cholesterol (LDL) and total cholesterol in one MR study^11^, whereas no causal associations with lipid levels were found in another^14^. One MR study evaluated blood pressure as an outcome, but no causal relationship was reported^12^. Furthermore, MR studies in African-ancestry populations have been lacking, despite marked differences in the distributions of many cardiometabolic traits across ancestries. As levels of circulating adiponectin have been found to be lower in African-ancestry individuals compared with European-ancestry individuals^16,17^, the effect of adiponectin on cardiometabolic disease risk could also differ. Furthermore, ethnic differences have been reported in adiponectin levels by weight status and in the association between adiponectin and cardiometabolic outcomes^18,19^.

We, therefore, performed MR analyses to assess the presence of a causal relationship between adiponectin and cardiometabolic outcomes, including insulin resistance, T2D, hypertension, blood pressure, HDL, LDL, total cholesterol, and triglycerides (TG) in sub-Saharan Africans. In addition, we assessed whether the association between adiponectin and cardiometabolic outcomes differed by overweight status and by sex.

## Methods

### Study population

The Africa America Diabetes Mellitus (AADM) study is a genetic epidemiology study of T2D in sub-Saharan Africa. T2D cases and controls were enrolled from university medical centers in Nigeria, Ghana, and Kenya^20,21^. Ethical approval was obtained for the AADM study from the National Institutes of Health and the ethical committees in Ghana (University of Ghana Medical School Research Ethics Committee and the Kwame Nkrumah University of Science and Technology Committee on Human Research Publication and Ethics), Nigeria (National Health Research Ethics Committee of Nigeria [NHREC]), and Kenya (The Moi Teaching & Referral Hospital / Moi University College of Health Sciences -Institutional Research and Ethics Committee [MTRH/MU-IREC]). All participants gave written informed consent prior to enrollment in the study. All procedures have been performed in accordance with the ethical standards as laid down in the 1964 Declaration of Helsinki and its later amendments or comparable ethical standards.

#### Genotyping

Genotyping was performed using either the Affymetrix Axiom PANAFR SNP array or the Illumina Multi-Ethnic Global Array (MEGA) as previously described^22^. Quality control was performed for each of the arrays separately, resulting in a sample-level genotype call rate of at least 0.95 for all samples. Imputation was performed using the African Genome Resources Haplotype Reference Panel via the Sanger Imputation Service.

#### Measurement of adiponectin

Adiponectin level was measured using multiplex-bead based flow cytometric immunoassays containing dyed microspheres linked with monoclonal antibodies specific for proteins in the plex according to the manufacturer’s instructions (Bioplex, Bio-Rad, Inc, Hercules, CA, USA). Adiponectin was included in the Bio-Plex Pro Human Diabetes Adipsin and Adiponectin Assays (Catalog #171A7002M). Circulating levels were determined using a Bio-Plex 200 array reader (Luminex, Austin, TX). Adiponectin concentrations were calculated using a standard curve with a manufacturer provided software, the Bio-Plex Manager Software. Adiponectin was log-transformed in all analyses.

#### Measurement of cardiometabolic outcomes and covariates

Demographic data, such as age and sex, were obtained through questionnaires and interviews. Participants were queried for their usual consumption frequency of beer, wine, gin, and liquor, which was recoded into any or no alcohol consumption. Participants who indicated having smoked cigarettes regularly for at least one year were considered current smokers and those who indicated having stopped smoking for at least one year after smoking regularly were considered former smokers. Current medication use was recorded and coded for blood pressure-lowering, glucose-lowering, or lipids medication. Physical exams were performed, and blood samples were collected by trained personnel during clinic visits. Height and weight were measured in light clothing and without shoes to the nearest 0.1 cm and kg using a stadiometer and electronic weighing scale. BMI was calculated as weight/height^2^ (kg/m^2^). Insulin resistance was determined using homeostatic modelling (HOMA) derived insulin resistance index (HOMA-IR)^23^. T2D status was determined using the American Diabetes Association (ADA) criteria which included a fasting plasma glucose of ≥ 7.0 mmol/L, a 2-h post load value of ≥ 11.1 (mmol/L) in the oral glucose tolerance test (OGTT) on more than one occasion or taking glucose-lowering medication as prescribed by a physician. HDL, LDL, total cholesterol, and TG were determined enzymatically with the COBAS Integra 400 Plus or Roche Modular-E Analyzer (Roche Diagnostics, Indianapolis, IN). Methods were standardized to in-house and other appropriate reference methods (e.g., CDC reference methods for HDL, isotope dilution mass spectrometry for total cholesterol and TG). Systolic blood pressure (SBP) and diastolic blood pressure (DBP) were measured twice in a sitting position with a ten-minute interval between measurements. The average of the first and second measurements was included in the analyses. Hypertension status was determined as SBP ≥ 130 mmHg, or DBP ≥ 80 mmHg, or being on antihypertensive medication^24^.

### Construction of the instrumental variable

**Figure 1:**
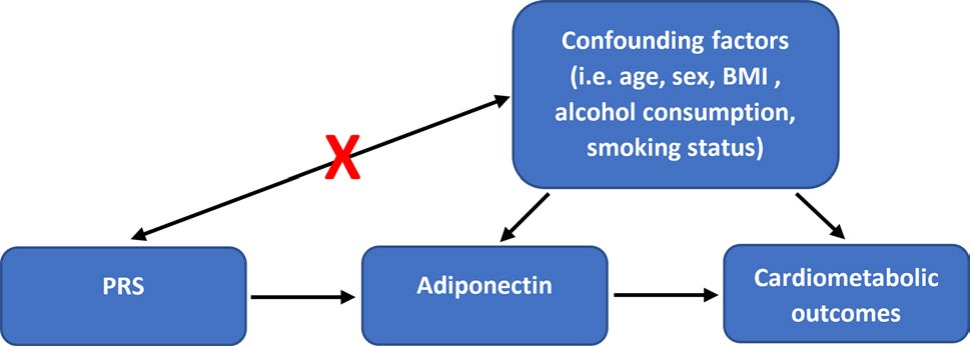
Conceptual model.

MR analyses were conducted using a PRS as an instrumental variable (Fig. 1). The use of PRS in MR analysis increases power and avoids weak instrument bias^25^. PRS are constructed by using weighted sums of risk alleles. Typically, these risk alleles are weighted using estimates from genome-wide association studies (GWAS) on the phenotype, usually a large meta-analysis. As large GWAS on adiponectin in sub-Saharan Africans are currently absent, we instead chose the largest meta-analysis for genome-wide association studies on adiponectin to date by Dastani et al.^11^ which included data from 16 GWAS comprising in total of 29,347 individuals of European ancestry. Summary statistics made available by the investigators of the ADIPOGen Consortium for the sex-combined meta-analysis (https://www.mcgill.ca/genepi/adipogen-consortium) were used.

Quality control (QC) of the AADM data and ADIPOGen summary statistics was performed following the guide to performing PRS analyses outlined by Choi et al*.* in *Nature Protocols*^26^. Only autosomes were retained. The AADM data were filtered for minor allele frequency (MAF) (< 0.01). Duplicated and ambiguous SNPs were removed. QC of the ADIPOGen summary statistics comprised of filtering for missingness per marker (> 0.01), missingness per sample (> 0.01), MAF (< 0.01), and Hardy–Weinberg equilibrium (*P*-value < 1 × 10^–6^). The quality of imputation was evaluated using INFO scores. Markers with INFO scores < 0.8 were removed from the AADM dataset. After filtering the genotyping rate was > 99%. Pruning was performed to remove highly correlated SNPs. We assessed heterozygosity rates and removed samples with an F coefficient that deviated more than three standard deviations (SD) from the mean. SNPs with mismatching alleles between AADM and ADIPOGen data were resolved by strand flipping and if that did not resolve the mismatch, they were excluded from the target data. We removed samples that had a first- or second-degree relative in the data. After filtering, there were 1,551,161 variants and 3393 samples in the final AADM dataset used for PRS construction.

The PRS was subsequently constructed by performing a clumping and thresholding (C + T) approach using the PRSice-2 software^27^. Clumping was performed to remove SNPs that are in Linkage Disequilibrium (LD) in order to retain SNPs that are largely independent of each other. The AADM data were used as LD reference panel. *P-*value thresholding was subsequently used to determine the optimized PRS by permuting the adiponectin values across the sample 10,000 times.

### Statistical analyses

#### Epidemiological analyses

Z-standardized log-transformed adiponectin values were used to evaluate the association with cardiometabolic outcomes per one SD increase. Continuous cardiometabolic outcomes were also converted to Z scores to make them comparable. The Z-standardized log-transformed adiponectin was tested for association with each of the cardiometabolic outcomes in partially- and fully adjusted models. Partially adjusted models were adjusted for age, sex, and population stratification by including the first three genetic principal components (PC). Fully adjusted models were adjusted for BMI, alcohol consumption, and smoking status in addition to the covariates of the partially adjusted model. Both partial and fully adjusted models were additionally adjusted for the use of blood pressure-lowering medication for SBP and DBP. Individuals on lipid-lowering medications (n = 39) were excluded from all analyses. All epidemiological analyses were performed using Stata 15.1 (Stata Corp, College Station, Texas).

#### Instrumental variable mendelian randomization analysis

The PRS was evaluated for core assumptions of an instrumental variable, including its association with adiponectin and potential confounders. The two-stage least-squares method implemented in the “ivregress” command in Stata 15.1 was used to estimate the causal effect of 1 SD increase in log-transformed adiponectin on each of the continuous cardiometabolic outcomes. For the binary traits, we predicted levels of adiponectin from linear regression analysis with the PRS and subsequently regressed these predicted values against the binary cardiometabolic outcomes of interest. We fitted partially and fully adjusted models, as defined in the epidemiological analyses. To assess the effect of weight status on the associations between adiponectin and cardiometabolic outcomes, analyses were stratified into normal weight individuals (BMI < 25 kg/m^2^) and overweight/obese individuals (BMI ≥ 25 kg/m^2^). As adiponectin levels display a strong sexual dimorphism^28^, analyses were stratified by sex as well.

#### Sensitivity analyses

The two-stage least-squares method makes parametric assumptions for the identification of causal effects. Hence, we used the semi-parametric two-step generalized method of moments (GMM) estimator to determine whether our results were method-dependent^29^. For continuous outcomes the GMM implemented in the “ivregress” command in Stata was used. For binary outcomes, the GMM Stata syntax as described by Clarke et al. was used^30^. Additionally, we conducted several sensitivity analyses to investigate potential pleiotropic effects, i.e. the influence of PRS genetic variants on the outcomes through other pathways than the exposure (adiponectin). First, we performed a series of regressions between the adiponectin PRS and age, sex, BMI, alcohol consumption, and smoking. Second, for each of the cardiometabolic outcomes, the SNP-adiponectin association betas were plotted against the SNP-outcome associations for the 222 genetic variants that compose the PRS. Summary statistics were derived by performing genome-wide regression analyses using the q.emmax test in *EPACTS* (version 3.2.6), which is a mixed model that can account for population structure and hidden relatedness^31^. Q.emmax is based on a linear model, and is useful for both quantitative and binary traits^31^. Possible pleiotropy was assessed visually with absence of outliers being supportive of homogeneity of causal estimates between genetic variants.

## Results

### Population characteristics

A total of 3,354 unrelated sub-Saharan Africans not using lipid-lowering medication were included in the present analyses. The study participants were 62% women, and the average age was 54.2 years (Table 1). The proportion consuming alcohol and currently smoking was relatively low. As expected for this sub-Saharan African population, a favorable lipid profile was observed across the sample. About half of the participants had T2D, because of the T2D case–control design of the AADM study.

**Table 1:** Characteristics of the study population^a^.

	Total (n = 3354)	Normal weight (n = 1251)	Overweight/obese (n = 2103)	*P*-value overweight/obese vs normal weight^b^
**Covariates**				
Age (years)	54.2 (53.8, 54.6)	54.2 (53.5, 54.9)	54.2 (53.7, 54.7)	0.976
Sex (% female)	62.4 (60.7, 64.0)	45.9 (43.1, 48.7)	72.2 (70.3, 74.1)	< 0.001
Alcohol intake (% yes)	27.2 (25.7, 28.8)	26.8 (24.4, 29.3)	27.5 (25.6, 29.4)	0.673
Smoking (% current)	3.7 (3.1, 4.4)	4.2 (3.3, 5.5)	3.3 (2.6, 4.2)	0.148
Smoking (% former)	11.6 (10.5, 12.7)	15.9 (13.9, 18.0)	9.0 (7.9, 10.3)	< 0.001
**Adiponectin**				
Adiponectin (ng/ml)	7,549 (4,097–17,403)	8,668 (4,749–19,389)	6,841 (3,827–16,493)	< 0.001
**Cardiometabolic outcomes**				
BMI (kg/m^2^)	27.4 (27.2, 27.6)	21.9 (21.8, 22.1)	30.7 (30.5, 31.0)	< 0.001
Obesity (%)	29.4 (27.8, 30.9)	NA	46.8 (44.7, 49.0)	NA
HOMA-IR^c^	1.23 (0.70 –2.20)	0.85 (0.49–1.55)	1.60 (0.92–2.61)	< 0.001
T2D (%)^d^	48.1 (46.4, 49.8)	44.1 (41.4, 46.7)	50.5 (48.4, 52.6)	< 0.001
HDL (mg/dl)	42.9 (42.3, 43.6)	45.7 (44.6, 46.8)	41.3 (40.5, 42.1)	< 0.001
LDL (mg/dl)	132.1 (130.4, 133.8)	127.9 (125.1, 130.8)	134.5 (132.5, 136.6)	0.0002
Total cholesterol (mg/dl)	203.3 (201.3, 205.3)	198.2 (194.7, 201.7)	206.4 (203.9, 208.8)	0.0001
Triglycerides (mg/dl)	98 (74–136)	90 (70–122)	102 (78–145)	< 0.001
SBP (mmHg)	138.2 (137.4, 139.0)	135.1 (133.8, 136.5)	140.0 (139.0, 141.0)	< 0.001
DBP (mmHg)	82.8 (82.4, 83.2)	79.9 (79.2, 80.6)	84.5 (84.0, 85.1)	< 0.001
Hypertension (%)	75.9 (74.5, 77.4)	66.8 (64.2, 69.4)	81.4 (79.6, 83.0)	< 0.001

*BMI* Body Mass Index, *T2D* Type 2 Diabetes, *SBP* Systolic Blood Pressure, *DBP* Diastolic Blood Pressure.

^a^Continuous variables are in means and corresponding (95% Confidence Intervals) for normally distributed variables. Non-normally distributed variables are expressed in medians and (25th–75th percentile). Categorical variables are in percentages with corresponding (95% Confidence Intervals).

^b^P-values were calculated using T-tests for differences in means, Mann–Whitney U tests for differences in medians, and chi square for differences in proportions.

^c^T2D cases were excluded for all HOMA-IR analyses.

^d^The high T2D prevalence is due to the T2D case–control study design of the AADM study.

Normal weight individuals (BMI < 25 kg/m^2^) were similar in age to overweight/obese individuals (BMI ≥ 25 kg/m^2^) and reported similar alcohol consumption and current smoking status, but they were more often male and former smokers (Table 1). Median adiponectin levels were lower in overweight/obese (6841 ng/ml) compared with normal weight individuals (8668 ng/ml). As expected, the prevalence of T2D and hypertension were higher in overweight/obese than in normal weight individuals and overweight/obese individuals had a less favorable lipid profile.

### Epidemiological association between adiponectin and cardiometabolic outcomes

In epidemiological analyses, adiponectin was significantly associated with all cardiometabolic outcomes evaluated in models adjusted for age, sex, population stratification, BMI, alcohol consumption, and smoking status (Table 2). Effect sizes were similar for the partial and fully adjusted models for all outcomes except SBP and DBP, which had larger effect sizes in the fully adjusted model. The addition of BMI as a covariate was responsible for most of this difference in effect sizes. One SD higher adiponectin levels were associated with lower HOMA-IR (β = -0.086), triglycerides (β = -0.141), and odds for T2D (OR = 0.81). In contrast, one SD higher adiponectin levels were associated with higher HDL (β = 0.162), LDL (β = 0.047), total cholesterol (β = 0.080), SBP (β = 0.049), DBP (β = 0.054), and hypertension (OR = 1.17).

**Table 2:** Associations between adiponectin and cardiometabolic outcomes^a^.

Cardiometabolic outcome	Partially adjusted^b^		Fully adjusted^c^	
Continuous^d^	Beta (95%CI)	P-value	Beta (95%CI)	P-value
HOMA-IR^e,f^	− 0.137 (− 0.181, − 0.093)	** < 0.001**	− 0.086 (− 0.128, − 0.044)	** < 0.001**
HDL	0.173 (0.137, 0.210)	** < 0.001**	0.162 (0.125, 0.200)	** < 0.001**
LDL	0.038 (0.001, 0.076)	**0.048**	0.047 (0.009, 0.085)	**0.016**
Total cholesterol	0.070 (0.032, 0.108)	** < 0.001**	0.080 (0.041, 0.118)	** < 0.001**
Triglycerides^f^	− 0.154 (− 0.190, − 0.119)	** < 0.001**	− 0.141 (− 0.177, − 0.106)	** < 0.001**
SBP	0.037 (0.004, 0.070)	**0.029**	0.049 (0.015, 0.083)	**0.005**
DBP	0.029 (− 0.007, 0.065)	0.111	0.054 (0.018, 0.091)	**0.003**

Binary^g^	OR (95%CI)	P-value	OR (95%CI)	P-value
T2D	0.80 (0.74, 0.86)	** < 0.001**	0.81 (0.74, 0.87)	** < 0.001**
Hypertension	1.08 (0.98, 1.18)	0.106	1.17 (1.07, 1.29)	**0.001**

^a^Values are presented per 1 SD increase in log transformed adiponectin.

^b^The partially adjusted model is adjusted for age, sex, and population stratification. SBP and DBP are additionally adjusted for blood pressure medication use.

^c^The fully adjusted model is adjusted for age, sex, population stratification, BMI, alcohol consumption, and smoking. SBP and DBP are additionally adjusted for blood pressure medication use.

^d^Values are betas with corresponding 95% Confidence Intervals.

^e^‡ T2D cases were excluded for all HOMA-IR analyses.

^f^HOMA-IR and Triglycerides were log transformed.

^g^Values are odds ratios with corresponding 95% Confidence Intervals.

### Adiponectin PRS

**Figure 2:**
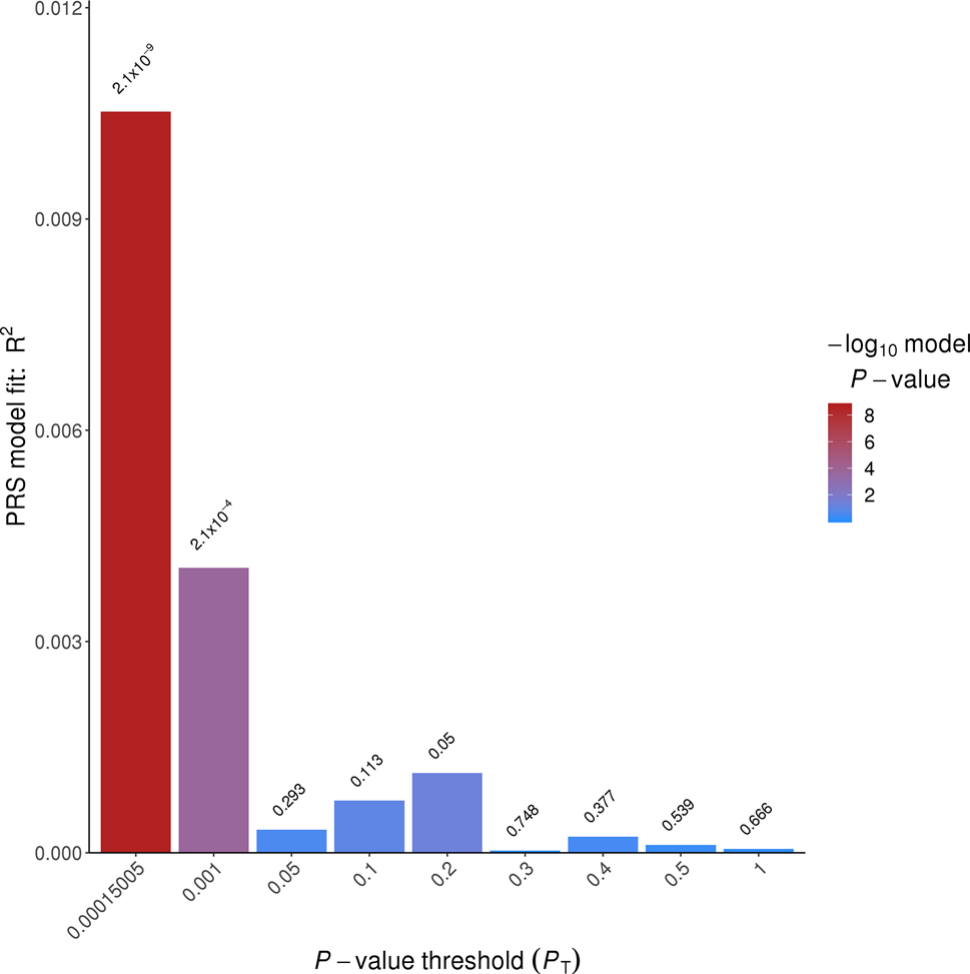
Bar plot of model fit of the adiponectin PRS at multiple P-value thresholds.

The adiponectin PRS with the best model fit contained 222 SNPs at a *P-*value threshold of 1.50 × 10^–4^ (Fig. 2). The GWAS summary statistics for these 222 SNPs are provided in Supplementary Table S1. Nineteen of these 222 SNPs were within or near (± 250 kb) the *ADIPOQ* gene, which codes for adiponectin. The PRS was confirmed to be a strong instrumental variable for adiponectin levels as defined by an F-statistic > 10^32^. The adiponectin PRS had an F statistic of 35.6 for its crude association with log-transformed adiponectin. When adjusted for age, sex, and population stratification the F-statistic was 116.7.

### Causal association between adiponectin and cardiometabolic outcomes

**Figure 3:**
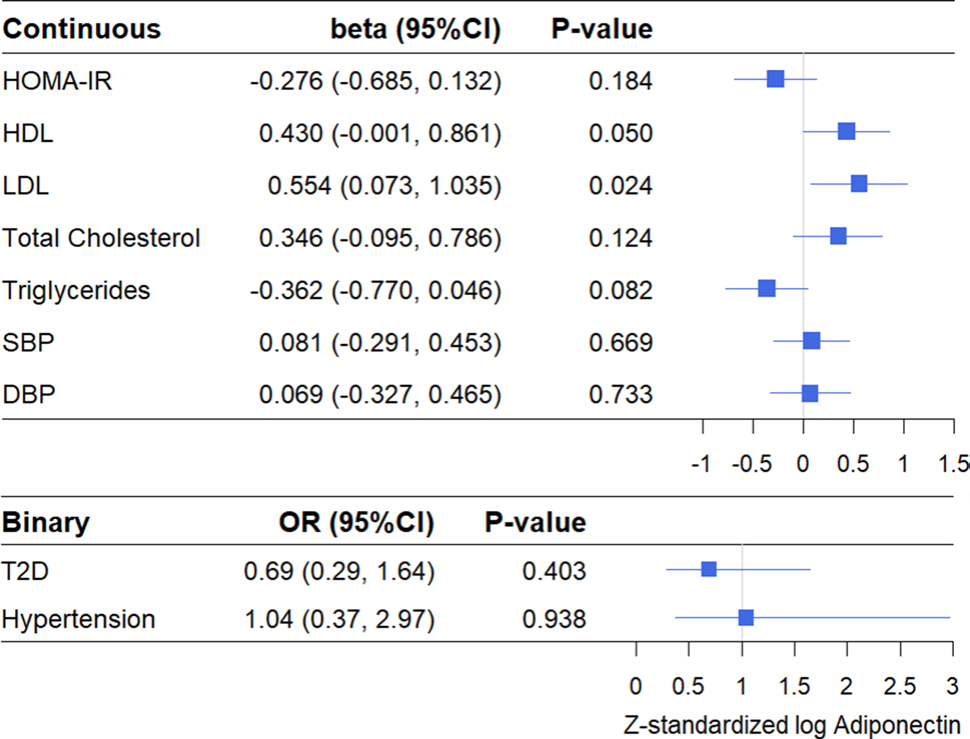
Causal associations between adiponectin and cardiometabolic outcomes using the two-stage least-squares method. Values are presented are adjusted for age, sex, population stratification, BMI, alcohol consumption, and smoking. SBP and DBP are additionally adjusted for blood pressure medication use. HOMA-IR and Triglycerides were log transformed. All continuous variables were Z-standardized. T2D cases were excluded for all HOMA-IR analyses.

**Figure 4:**
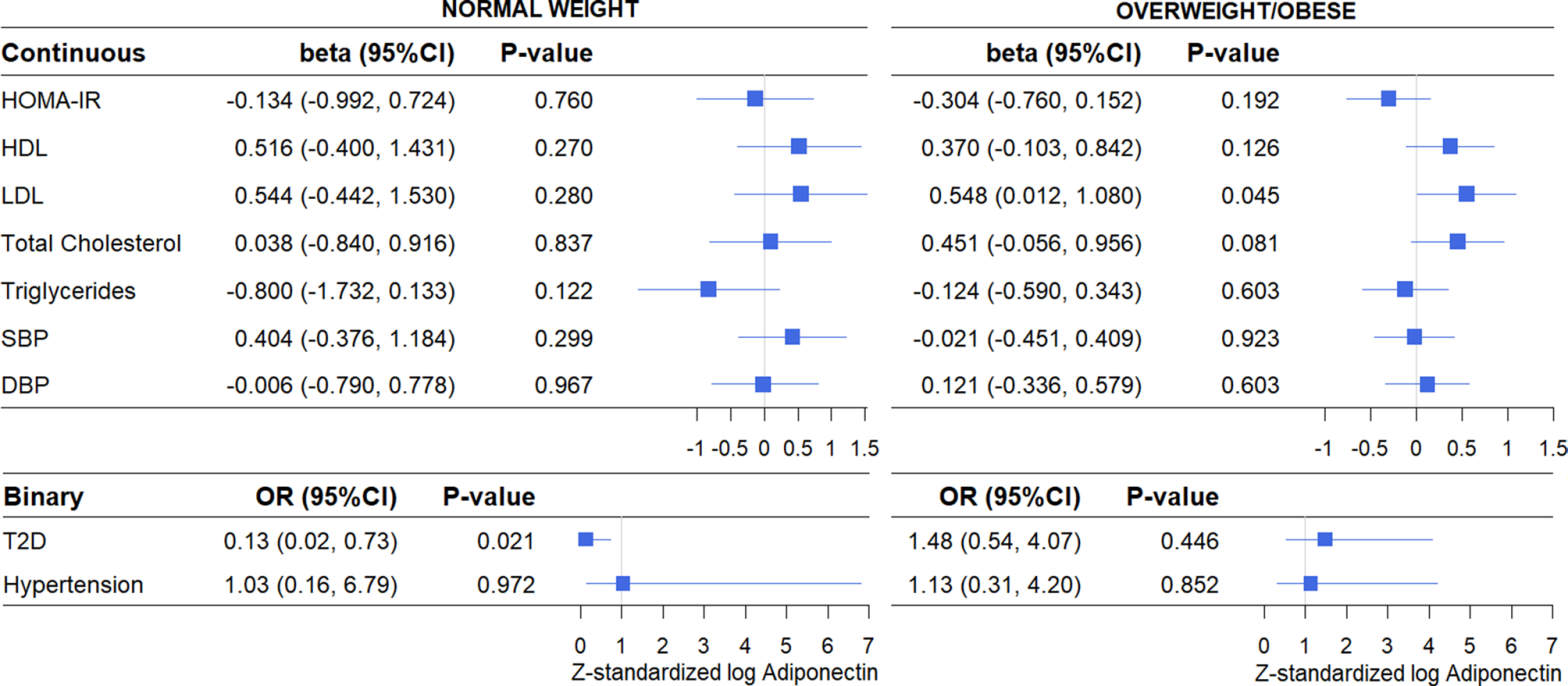
Causal associations between adiponectin and cardiometabolic outcomes in normal weight and overweight/obese individuals using the two-stage least-squares method. Values are presented are adjusted for age, sex, population stratification, BMI, alcohol consumption, and smoking. SBP and DBP are additionally adjusted for blood pressure medication use. HOMA-IR and Triglycerides were log transformed. All continuous variables were Z-standardized. T2D cases were excluded for all HOMA-IR analyses.

**Figure 5:**
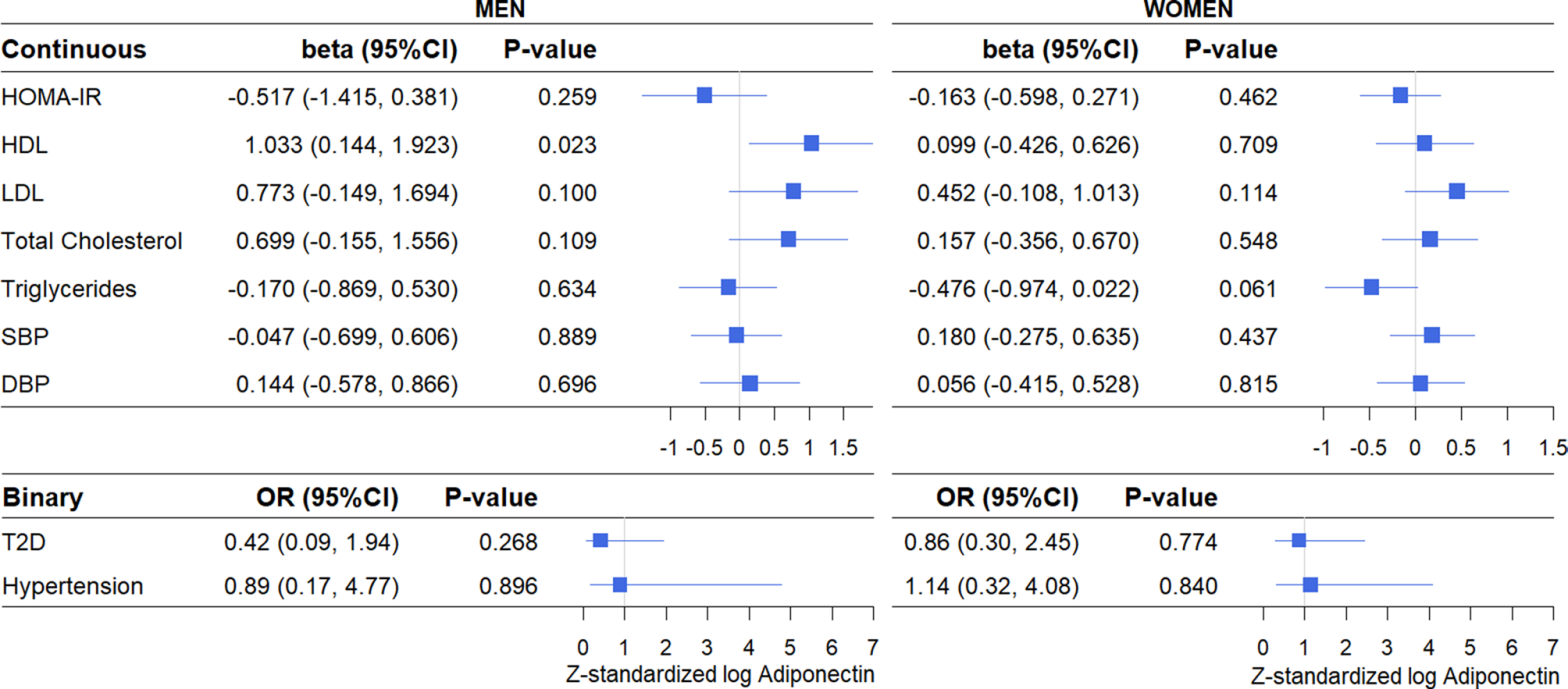
Causal associations between adiponectin and cardiometabolic outcomes in men and women using the two-stage least-squares method. Values are presented are adjusted for age, population stratification, BMI, alcohol consumption, and smoking. SBP and DBP are additionally adjusted for blood pressure medication use. HOMA-IR and Triglycerides were log transformed. All continuous variables were Z-standardized. T2D cases were excluded for all HOMA-IR analyses.

Genetically elevated levels of adiponectin (i.e., adiponectin PRS) were not associated with HOMA-IR, HDL, total cholesterol, triglycerides, SBP, DBP, T2D, or hypertension (Fig. 3). The only statistically significant association was for LDL with genetically elevated adiponectin levels associated with higher LDL levels in both the fully adjusted model (β 0.55, 95%CI 0.07–1.04, *P-*value 0.024) (Fig. 3) and in the partially adjusted model (β 0.54, 95%CI 0.07–1.01, *P-*value 0.025) (Supplementary Figure S1). The effect size was similar for both models. In models stratified for weight status, the positive association between genetically elevated adiponectin and LDL remained only in overweight/obese individuals (β 0.55, 95%CI 0.01–1.08, *P-*value 0.045) (Fig. 4). In normal weight individuals, genetically elevated adiponectin was associated with lower odds for T2D in both the fully adjusted model (OR 0.13, 95%CI 0.02–0.73, *P-*value 0.021) (Fig. 4) and the partially adjusted model (OR 0.16, 95%CI 0.03–0.81, *P-*value 0.027) (Supplementary Figure S2). There was no evidence for a causal association between adiponectin and any of the other cardiometabolic outcomes in either normal weight or overweight/obese individuals. In sex stratified analyses, genetically elevated adiponectin was associated with higher HDL levels in men (β 1.03, 95%CI 0.14–1.92, *P-*value 0.023), but not in women (β 0.10, 95%CI − 0.43 to 0.63, *P-*value 0.709) (Fig. 5). None of the other cardiometabolic outcomes were associated with the adiponectin PRS in men and women separately. Sex-stratified findings were similar for the partially adjusted model (Supplementary Figure S3). Overall, epidemiological and causal estimates were directionally concordant.

### Sensitivity analyses

**Figure 6:**
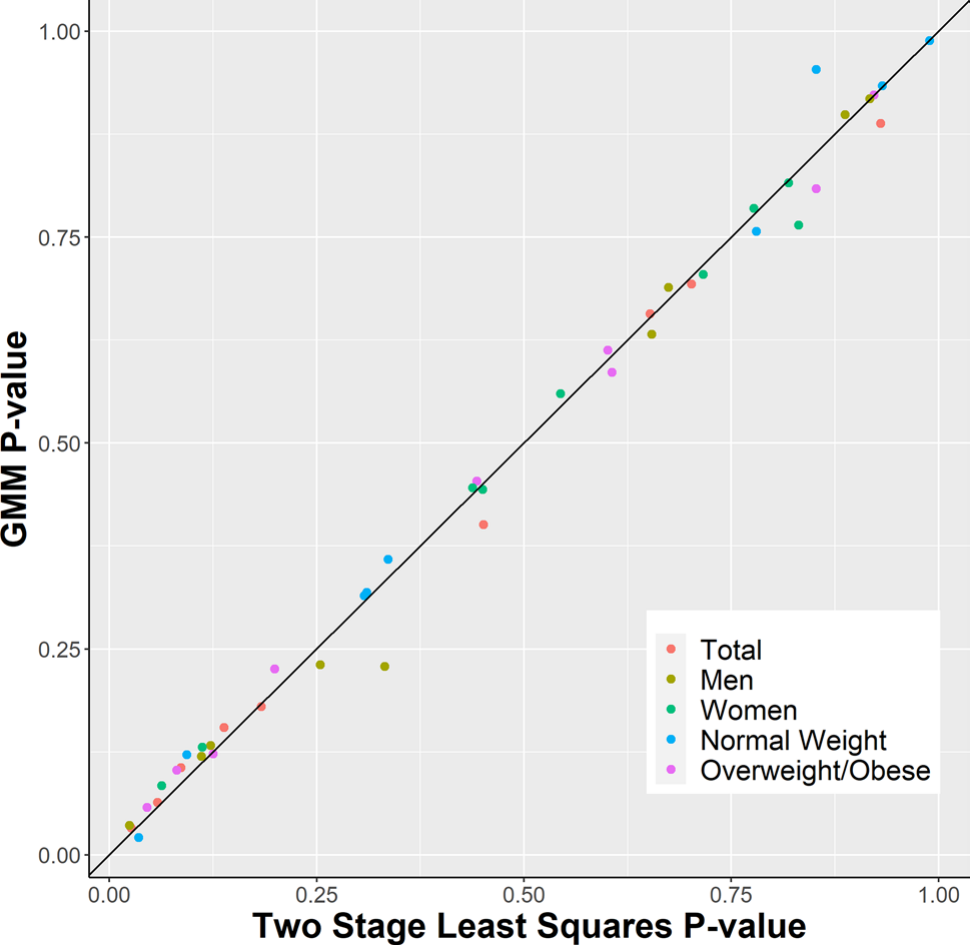
Concordance between P-values of two-stage least-squares method and generalized method of moments (GMM) for all models.

The generalized method of moments (GMM) estimator generated similar causal estimates as the two-stage least-squares method with high concordance between P-values of both methods (Fig. 6). A side-by-side comparison of both MR methods shows that the adiponectin PRS remained causally associated with only LDL in the total sample in both fully adjusted models (β 0.55, 95%CI 0.05–1.06, *P-*value 0.031) (Supplementary Figure S4) and partially adjusted models (β 0.54, 95%CI 0.05–1.03, *P*-value 0.031) (Supplementary Figure S1). In overweight/obese individuals, the PRS was still associated with higher LDL in the partially adjusted model (β 0.57, 95%CI 0.01–1.13, *P*-value 0.046) (Supplementary Figure S2) but lost statistical significance in the fully adjusted model (β 0.55, 95%CI − 0.02 to 1.12, *P*-value 0.058) (Supplementary Figure S5). The association between the adiponectin PRS and lower odds for T2D in normal weight individuals remained in both models (fully adjusted OR 0.58, 95%CI 0.37–0.92, *P*-value = 0.021). The two-stage least squares and GGM method also demonstrated high concordance for the sex stratified analyses, with the causal association between the adiponectin PRS and higher HDL in men only in both fully (Supplementary Figure S6) and partially adjusted models (Supplementary Figure S3). Furthermore, we did not find evidence for confounding of our causal estimates, as the adiponectin PRS was not associated with age (*P-*value = 0.09), sex (*P-*value = 0.64), BMI (*P-*value = 0.23), alcohol consumption (*P-*value = 0.57), or smoking (*P-*value = 0.87). No outliers were observed in scatter plots of SNP-adiponectin associations against SNP-outcome associations (Supplementary Figure S7).

## Discussion

Genetically elevated adiponectin concentration as estimated using a PRS as an instrumental variable was associated with LDL in the total sample of sub-Saharan Africans. In analyses stratified by weight status, the adiponectin PRS was associated with LDL in overweight/obese individuals and with T2D in normal weight individuals. In sex stratified analyses, the adiponectin PRS was associated with HDL in men. The PRS was not associated with any of the other cardiometabolic outcomes. These findings were robust in sensitivity analyses.

This is the first MR study to report evidence for a causal association between adiponectin and LDL. Previous adiponectin MR studies that evaluated LDL as an outcome did not find evidence to support a causal association^11,14,33^. Only one of these three studies included African ancestry populations in their LDL MR analyses and they made up merely 7% of their multi-ethnic study sample^14^. Also, the African-ancestry populations included were African Americans who differ in both environmental and genetic background from the sub-Saharan Africans in the present analyses. Ethnic differences between study populations may therefore play a role in the difference in findings.

The observed associations were not in a consistent direction in terms of favorable vs. unfavorable cardiometabolic outcomes. Higher adiponectin levels were associated with higher circulating LDL among sub-Saharan Africans in both epidemiological and causal association analyses. The majority of previous epidemiological studies reported no association between adiponectin and LDL concentration^34–36^, including studies in African-ancestry individuals^37,38^, with only a few studies reporting an inverse relationship ^39,40^. Based on these epidemiological studies, adiponectin was not thought to play a role in the regulation of LDL concentration^41^. Our MR analyses suggest that the potential effect of adiponectin levels on LDL concentration may need to be revisited. Although adiponectin has anti-inflammatory and cardiovascular protective properties in healthy subjects, it is increasingly recognized that its beneficial effects alter under metabolically unfavorable conditions^42^. Adiponectin’s anti-inflammatory effects and favorable associations with most cardiometabolic outcomes have largely been observed in healthy populations or those with a very early disease state. Adiponectin has been reported as a predictor of all-cause and cardiovascular mortality in individuals aged 50 to 75 in whom cardiovascular diseases were prevalent^43^ and causally associated with cardiovascular mortality in T2D patients^44^. This apparent contradictory relationship has been described as the “adiponectin paradox”^45–47^. It has been hypothesized that adiponectin protects against cardiometabolic diseases in healthy individuals but is compensatorily up-regulated in patients already afflicted with cardiometabolic diseases^43^. Furthermore, both high and low LDL are predictors of cardiovascular mortality^48,49^. Further studies are needed to investigate under which specific metabolic conditions adiponectin causally affects LDL concentration and what the underlying mechanisms are for this relationship.

Our findings support an inverse causal effect of adiponectin on T2D in normal weight individuals alone. Previous large MR studies have reported conflicting results with some finding evidence for a causal effect on T2D^11^ and others finding no evidence for such causality^14,15^. None of these MR studies assessed the effect of overweight status. Nevertheless, a differential effect of adiponectin by overweight status has been reported previously^42^. Studies have shown that adiponectin concentration and adiponectin expression are higher in normal weight individuals^50^. Accordingly, we observed higher median adiponectin levels in normal weight individuals compared with overweight/obese. It has been proposed that an inflammatory response resulting from increased adiposity can lead to a reduction in adiponectin^51^. A longitudinal study in European- and African Americans found associations between adiponectin and T2D incidence only in those with low systemic inflammation^52^. Adiponectin’s protective effects for T2D development may be less effective in a state of chronic inflammation caused by high adiposity, which is thought to be partially explained by adiponectin resistance, a condition usually seen along with insulin resistance and a decline in adiponectin receptor (AdipoR1/R2) mRNA expression.

The role of adiponectin in T2D risk may differ by ancestry and/or ethnicity. Normal weight sub-Saharan African migrants have been found to have a disproportionately high prevalence of T2D compared with European-ancestry populations^6^. In rural Ghana, T2D in normal weight individuals accounted for 56% of all T2D cases^5^. A previous study reported lower adiponectin levels in normal weight African women compared with European-ancestry women, while adiponectin levels did not differ in overweight/obese women^16^. It is currently unclear how the relative roles of genetic versus non-genetic factors may explain these differences. In a study of African Americans, the proportion of European ancestry was directly associated with adiponectin in lay healthy individuals, but not in obese or insulin resistant individuals, suggesting an interaction between genetic and metabolic factors^53^.

We found a differential effect of adiponectin on HDL between men and women with evidence for a causal effect on higher HDL levels only in men. Sex differences in the association between adiponectin and HDL have been reported previously. A recent prospective study in 1,110 Korean men and women aged 40 to 70 found an independent association between adiponectin levels and risk of incident metabolic syndrome in men but not in women^54^. HDL and blood pressure were the metabolic syndrome components that drove this association. There is strong sexual dysmorphia in adiponectin with on average higher circulating levels in women than in men^28^. While differences in hormonal regulation^28^ and visceral adipose tissue^55^ may explain part of this dysmorphia, other factors determining the sex difference remain to be elucidated.

A key strength of this study is that we used a PRS as an instrumental variable in the MR analyses. PRS have been shown to have advantages over using single SNPs or non-weighted multi-SNP instrumental variables^25^. Another strength is that LDL was directly assayed rather than estimated using the widely used Friedewald equation, which is less accurate. Thirdly, we validated our MR findings using another statistical method to confirm that our causal estimates are robust to the analytical approach used. Nevertheless, our study is not without limitations. Sub-Saharan Africans are greatly underrepresented in GWAS^56^ and thus no GWAS meta-analysis was available to use as base data for the PRS computation. Because of this, individual SNPs that compose the PRS were not genome-wide significantly associated with adiponectin in sub-Saharan Africans. This prevented us from running alternative MR methods that require individual SNP associations, such as MR Egger analyses. Nevertheless, our PRS was strongly associated with adiponectin in our study population and sensitivity analyses using the PRS rather than individual SNP associations generated similar findings to our main analyses. The lack of diversity in genomics studies has also hampered our ability to replicate our findings in another sub-Saharan African sample. There is a need for more sub-Saharan African cohorts that include genotyping data as well as measurement of adipocytokines. Lastly, we used total adiponectin in this study, while high-molecular weight adiponectin is the most biologically active form of adiponectin in terms of glucose homeostasis. Nevertheless, previous studies have shown similar associations with T2D incidence and metabolic parameters for total and high-molecular weight adiponectin^57,58^.

The findings of this MR analysis in sub-Saharan Africans support a causal effect of adiponectin concentration on LDL, on T2D in normal weight individuals, and on HDL in men. Our results do not provide evidence for a causal association with other cardiometabolic outcomes in sub-Saharan Africans. Differences between our findings and findings from previous MR, consisting largely of European-ancestry populations, could be due to ethnic differences in adiponectin biology or due to differences in metabolic health of the study populations. Large meta-analyses that include sub-Saharan Africans are needed to be able to better infer causality in relationships between adipocytokines and cardiometabolic outcomes in African-ancestry populations.

## Supplementary Information

Supplementary Information.

## Data Availability

The AADM dataset analyzed in the current study is available from the senior corresponding author upon reasonable request by e-mail (rotimic@mail.nih.gov) as part of a collaboration. These data are not available through a repository due to the consent obtained which does not grant permission for deposition.
