# Supplementary material for: Mendelian randomization study reveals a causal relationship between adiponectin and LDL cholesterol in Africans
Source: Sci Rep. 2022 Nov 8;12:18955. doi: 10.1038/s41598-022-21922-w (PMC9643497; doi:10.1038/s41598-022-21922-w)
Supplement: Supplementary file 1 — Supplementary Information. [file 41598_2022_21922_MOESM1_ESM.pdf]

SUPPLEMENTARY MATERIAL

Manuscript Title: Mendelian Randomization Study Reveals a Causal Relationship between Adiponectin and LDL Cholesterol in Africans

Authors: Karlijn A.C. Meeks, Amy R. Bentley, Ayo P. Doumatey, Adebowale A. Adeyemo, Charles N. Rotimi

Contents:

Figure S1. Causal associations between adiponectin and cardiometabolic outcomes for partially adjusted models using the two-stage least-squares method (blue) and the Generalized Method of Moments (red).

Figure S2. Causal associations between adiponectin and cardiometabolic outcomes in normal weight and overweight/obese individuals for partially adjusted models using the two-stage least-squares method (blue) and the Generalized Method of Moments (red).

Figure S3. Causal associations between adiponectin and cardiometabolic outcomes in men and women for partially adjusted models using the two-stage least-squares method (blue) and the Generalized Method of Moments (red).

Figure S4. Side-by-side comparison of the two-stage least-squares method (blue) and the Generalized Method of Moments (red) for the fully adjusted causal associations between adiponectin and cardiometabolic outcomes.

Figure S5. Side-by-side comparison of the two-stage least-squares method (blue) and the Generalized Method of Moments (red) for the fully adjusted causal associations between adiponectin and cardiometabolic outcomes in normal weight and overweight/obese individuals.

Figure S6. Side-by-side comparison of the two-stage least-squares method (blue) and the Generalized Method of Moments (red) for the fully adjusted causal associations between adiponectin and cardiometabolic outcomes in men and women.

Figure S7. Scatter plots of SNP-adiponectin associations against SNP-outcome associations for the PRS SNPs.

Table S1. GWAS summary statistics for the adiponectin PRS comprising SNPs.

Continuous	beta (95%CI)	P-value
HOMA-IR	-0.340 (-0.759, 0.080)	0.112
	-0.340 (-0.758, 0.078)	0.111
HDL	0.399 (-0.019, 0.817)	0.062
	0.399 (-0.028, 0.826)	0.067
LDL	0.540 (0.067, 1.012)	0.025
	0.540 (0.050, 1.030)	0.031
Total Cholesterol	0.344 (-0.086, 0.775)	0.117
	0.344 (-0.102, 0.790)	0.130
Triglycerides	-0.316 (-0.713, 0.081)	0.118
	-0.316 (-0.736, 0.103)	0.140
SBP	0.040 (-0.322, 0.402)	0.828
	0.040 (-0.327, 0.407)	0.831
DBP	0.025 (-0.363, 0.413)	0.901
	0.025 (-0.350, 0.399)	0.898

Binary	OR (95%CI)	P-value
T2D	0.71 (0.31, 1.63)	0.421
	0.79 (0.47, 1.34)	0.384
Hypertension	1.07 (0.40, 2.90)	0.890
	1.02 (0.82, 1.25)	0.882

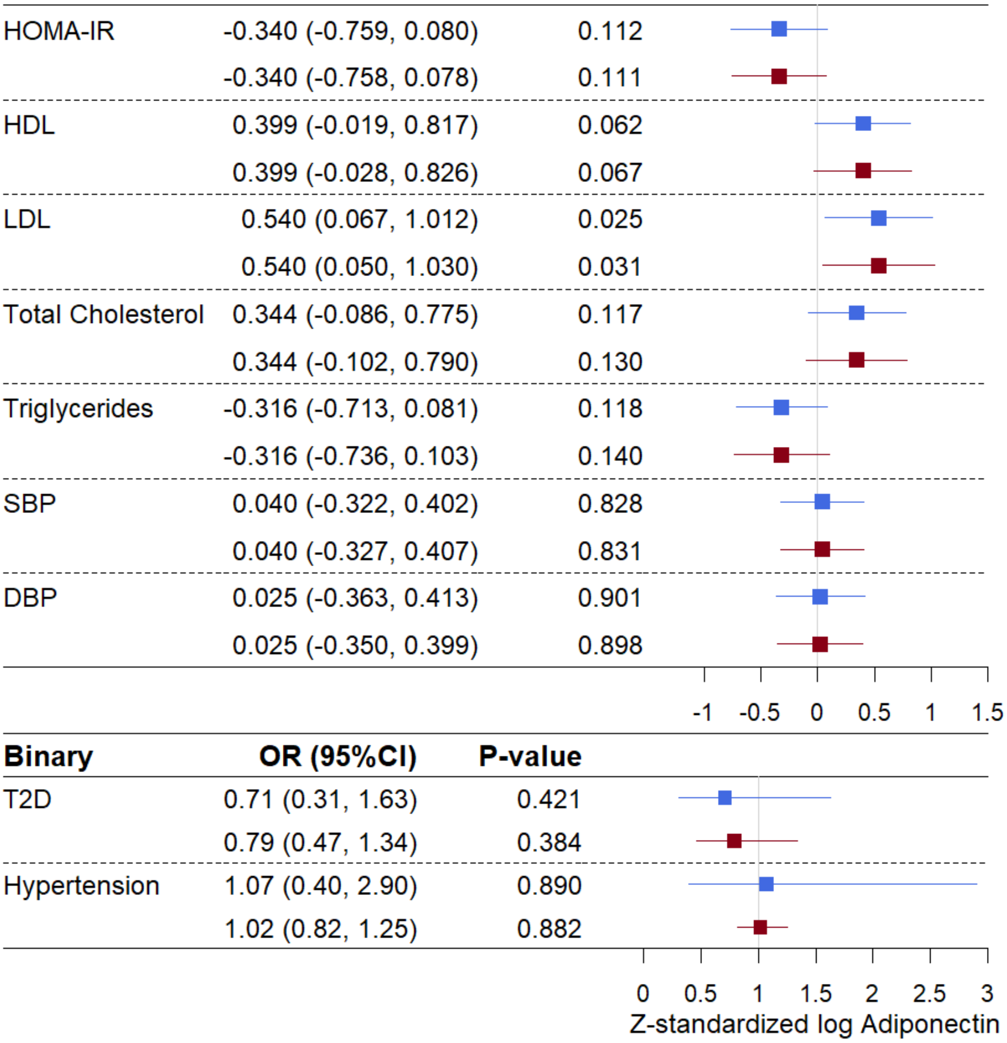

Figure S1. Causal associations between adiponectin and cardiometabolic outcomes for partially adjusted models using the two-stage least-squares method (blue) and the Generalized Method of Moments (red).

Values are presented are adjusted for age, sex, and population stratification. SBP and DBP are additionally adjusted for blood pressure medication use. HOMA-IR and Triglycerides were log transformed. All continuous variables were Z-standardized. T2D cases were excluded for all HOMA-IR analyses.

Continuous	NORMAL WEIGHT beta (95%CI)	P-value	OVERWEIGHT/OBESE beta (95%CI)	P-value
HOMA-IR	-0.230 (-1.164, 0.705)	0.630	-0.259 (-0.699, 0.181)	0.249
	-0.230 (-1.051, 0.591)	0.583	-0.259 (-0.724, 0.207)	0.276
HDL	0.448 (-0.415, 1.310)	0.309	0.342 (-0.117, 0.801)	0.144
	0.448 (-0.451, 1.346)	0.329	0.342 (-0.116, 0.800)	0.143
LDL	0.442 (-0.513, 1.140)	0.364	0.572 (0.042, 1.102)	0.034
	0.442 (-0.525, 1.408)	0.370	0.572 (0.010, 1.134)	0.046
Total Cholesterol	0.040 (-0.831, 0.910)	0.929	0.469 (-0.025, 0.963)	0.063
	0.040 (-0.851, 0.930)	0.930	0.469 (-0.059, 0.997)	0.081
Triglycerides	-0.703 (-1.579, 0.174)	0.116	-0.118 (-0.573, 0.338)	0.613
	-0.703 (-1.637, 0.232)	0.141	-0.118 (-0.590, 0.355)	0.626
SBP	0.362 (-0.382, 1.105)	0.340	-0.088 (-0.512, 0.337)	0.686
	0.362 (-0.402, 1.125)	0.353	-0.088 (-0.518, 0.343)	0.690
DBP	-0.048 (-0.797, 0.702)	0.901	0.064 (-0.384, 0.511)	0.780
	-0.048 (-0.812, 0.717)	0.903	0.064 (-0.360, 0.487)	0.768

Binary	NORMAL WEIGHT OR (95%CI)	P-value	OVERWEIGHT/OBESE OR (95%CI)	P-value
T2D	0.16 (0.03, 0.81)	0.027	1.51 (0.57, 4.01)	0.409
	0.58 (0.37, 0.91)	0.019	1.24 (0.75, 2.04)	0.397
Hypertension	0.95 (0.16, 5.59)	0.957	1.24 (0.35, 4.44)	0.736
	0.98 (0.51, 1.88)	0.949	1.04 (0.85, 1.27)	0.718

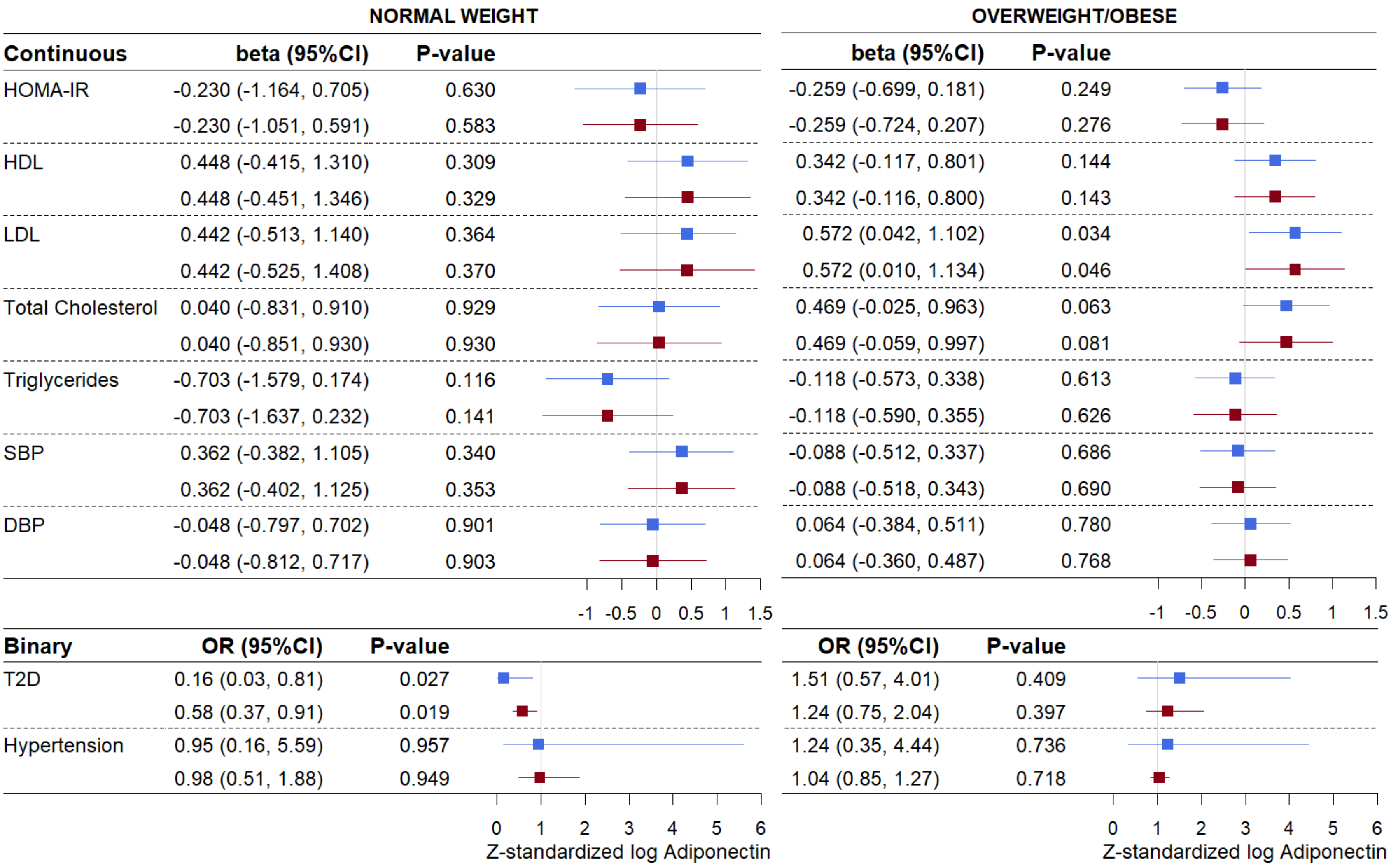

Figure S2. Causal associations between adiponectin and cardiometabolic outcomes in normal weight and overweight/obese individuals for partially adjusted models using the two-stage least-squares method (blue) and the Generalized Method of Moments (red).

Values are presented are adjusted for age, sex, and population stratification. SBP and DBP are additionally adjusted for blood pressure medication use. HOMA-IR and Triglycerides were log transformed. All continuous variables were Z-standardized. T2D cases were excluded for all HOMA-IR analyses.

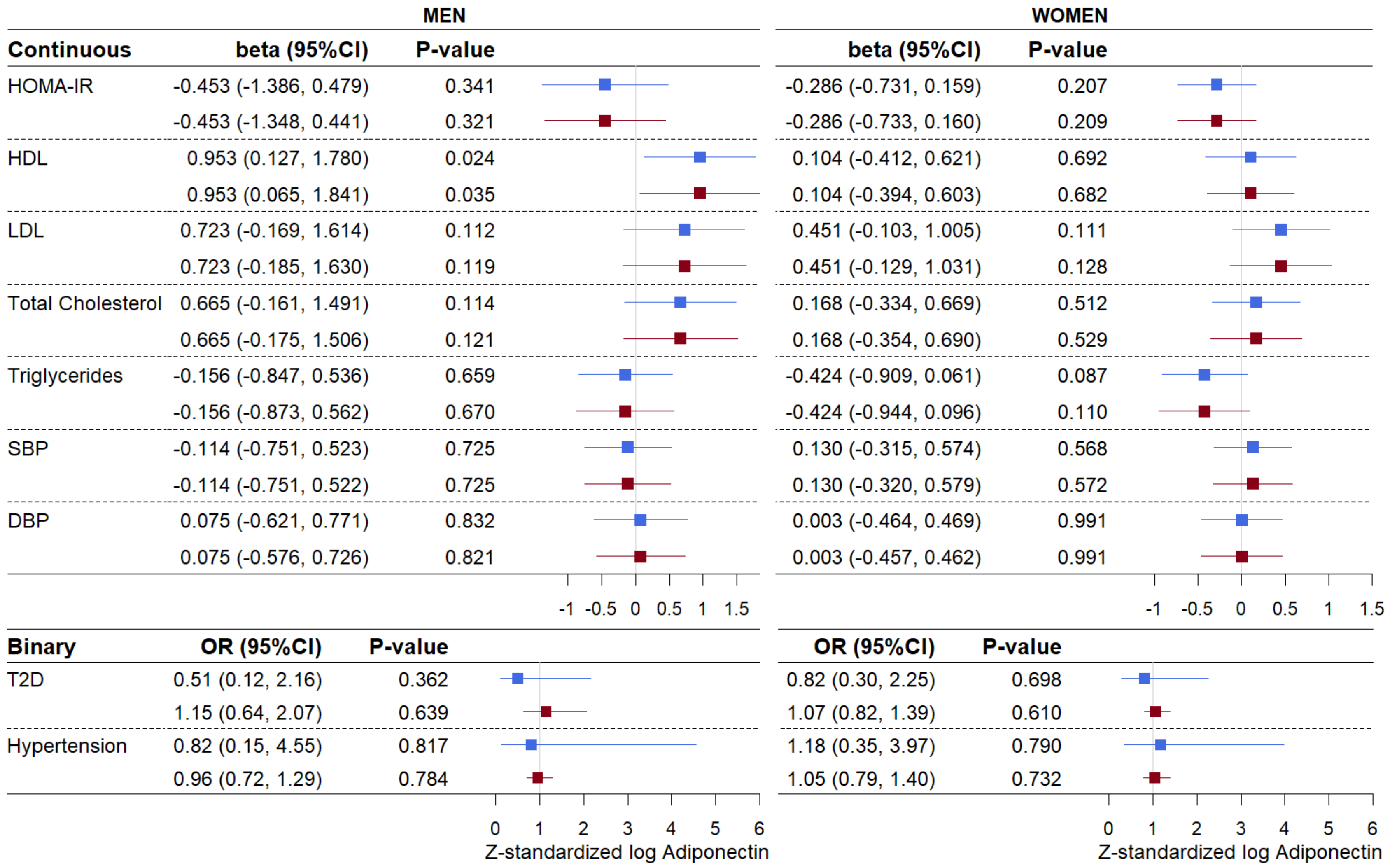

Figure S3. Causal associations between adiponectin and cardiometabolic outcomes in men and women for partially adjusted models using the two-stage least-squares method (blue) and the Generalized Method of Moments (red).

Values are presented are adjusted for age and population stratification. SBP and DBP are additionally adjusted for blood pressure medication use. HOMA-IR and Triglycerides were log transformed. All continuous variables were Z-standardized. T2D cases were excluded for all HOMA-IR analyses.

Continuous	beta (95%CI)	P-value
HOMA-IR	-0.276 (-0.685, 0.132)	0.184
	-0.276 (-0.682, 0.129)	0.181
HDL	0.430 (-0.001, 0.861)	0.050
	0.430 (-0.012, 0.871)	0.056
LDL	0.554 (0.073, 1.035)	0.024
	0.554 (0.051, 1.056)	0.031
Total Cholesterol	0.346 (-0.095, 0.786)	0.124
	0.346 (-0.114, 0.805)	0.140
Triglycerides	-0.362 (-0.770, 0.046)	0.082
	-0.362 (-0.795, 0.071)	0.101
SBP	0.081 (-0.291, 0.453)	0.669
	0.081 (-0.297, 0.459)	0.674
DBP	0.069 (-0.327, 0.465)	0.733
	0.069 (-0.314, 0.4533)	0.725

Binary	OR (95%CI)	P-value
T2D	0.69 (0.29, 1.64)	0.403
	0.80 (0.47, 1.37)	0.417
Hypertension	1.04 (0.37, 2.97)	0.938
	1.02 (0.81, 1.27)	0.892

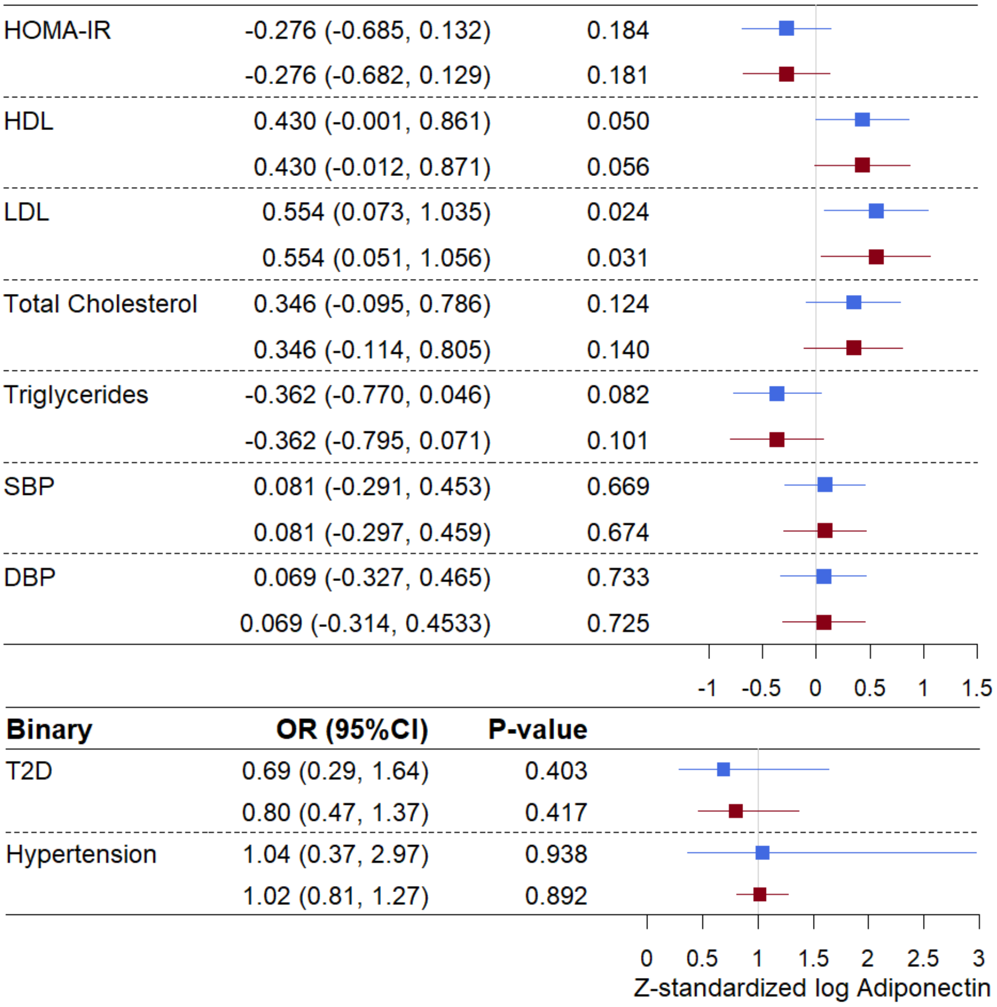

Figure S4. Side-by-side comparison of the two-stage least-squares method (blue) and the Generalized Method of Moments (red) for the fully adjusted causal associations between adiponectin and cardiometabolic outcomes.

Values are presented are adjusted for age, sex, population stratification, BMI, alcohol consumption, and smoking. SBP and DBP are additionally adjusted for blood pressure medication use. HOMA-IR and Triglycerides were log transformed. All continuous variables were Z-standardized. T2D cases were excluded for all HOMA-IR analyses.

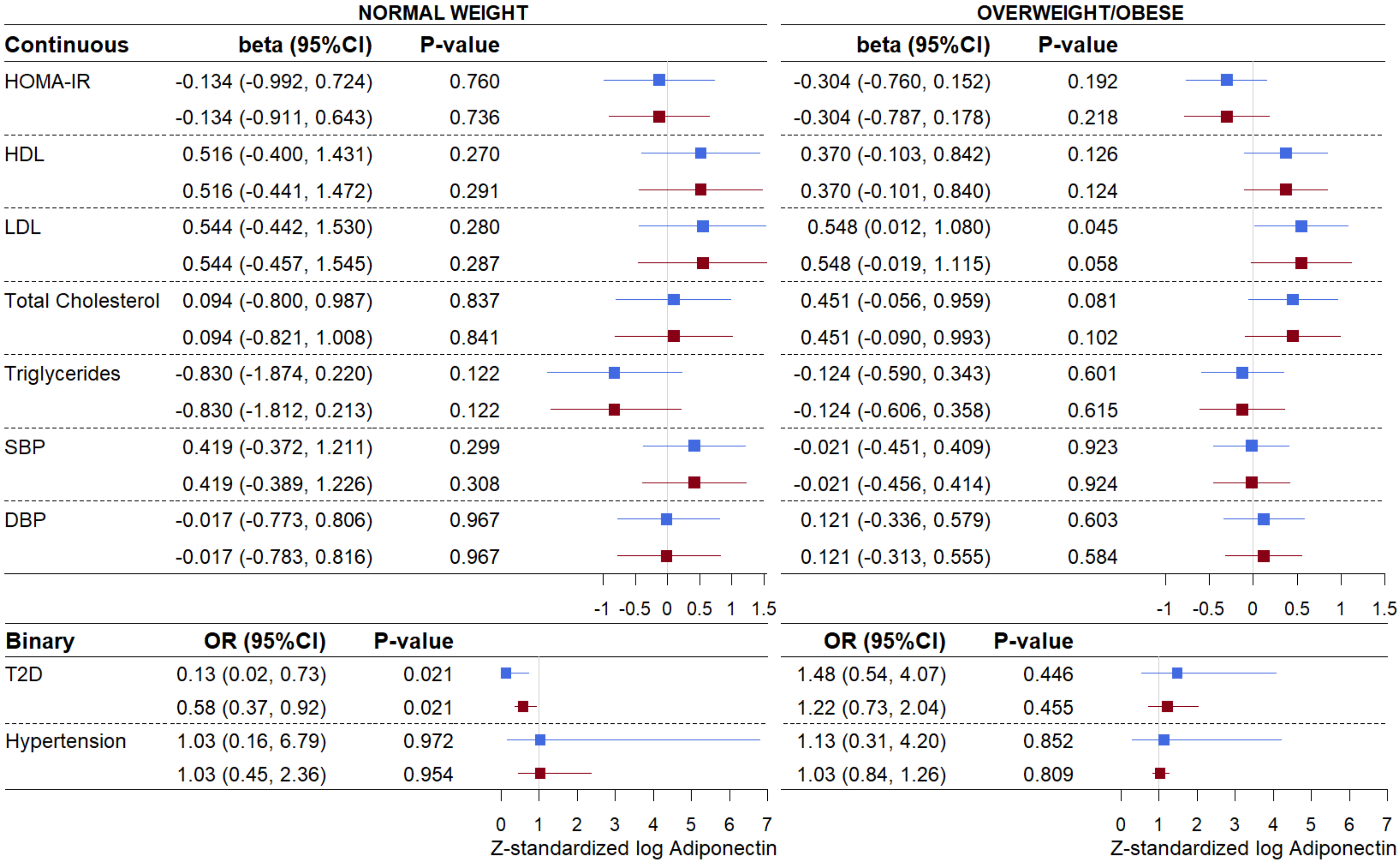

Figure S5. Side-by-side comparison of the two-stage least-squares method (blue) and the Generalized Method of Moments (red) for the fully adjusted causal associations between adiponectin and cardiometabolic outcomes in normal weight and overweight/obese individuals.

Values are presented are adjusted for age, sex, population stratification, BMI, alcohol consumption, and smoking. SBP and DBP are additionally adjusted for blood pressure medication use. HOMA-IR and Triglycerides were log transformed. All continuous variables were Z-standardized. T2D cases were excluded for all HOMA-IR analyses.

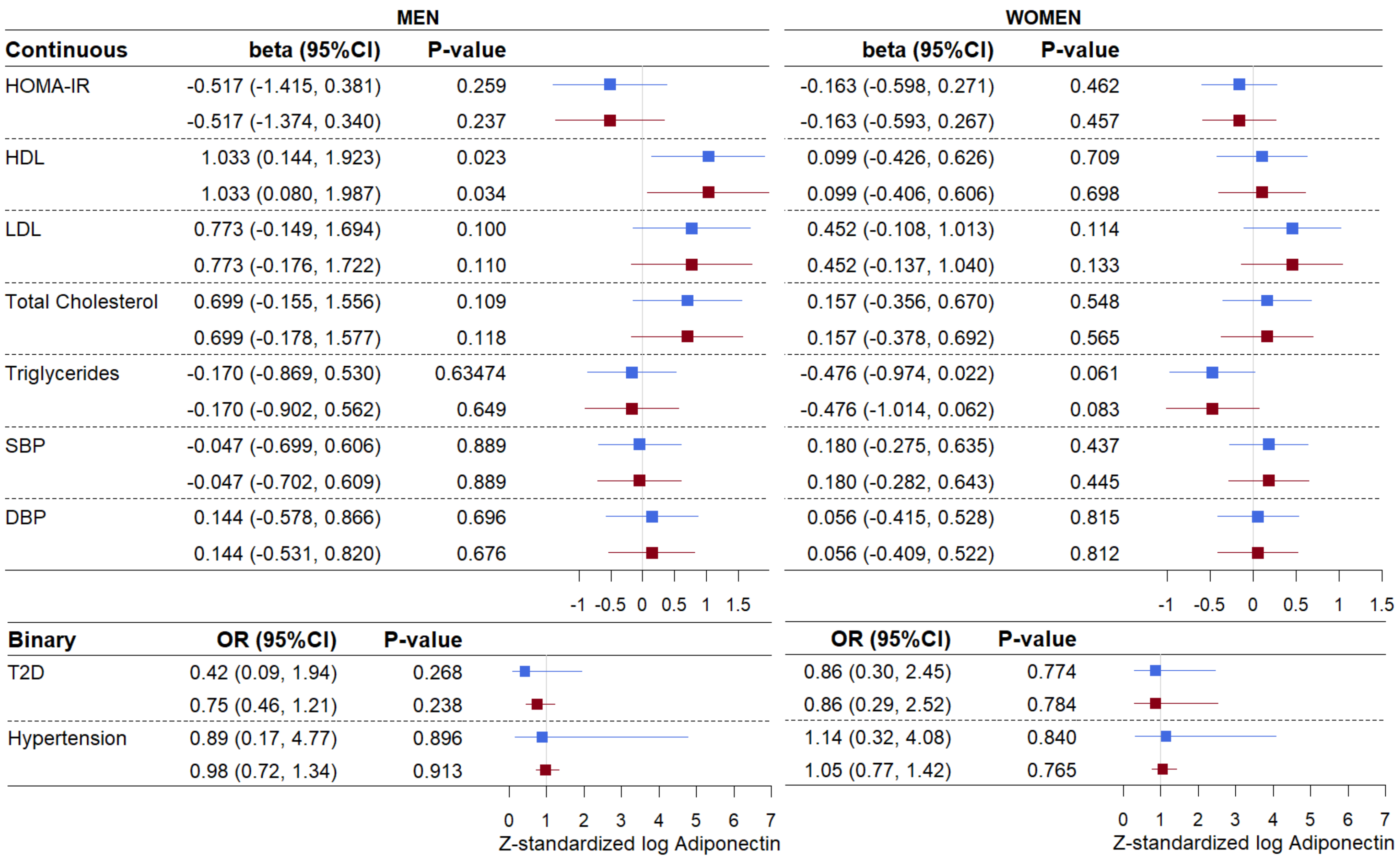

Figure S6. Side-by-side comparison of the two-stage least-squares method (blue) and the Generalized Method of Moments (red) for the fully adjusted causal associations between adiponectin and cardiometabolic outcomes in men and women.

Values are presented are adjusted for age, population stratification, BMI, alcohol consumption, and smoking. SBP and DBP are additionally adjusted for blood pressure medication use. HOMA-IR and Triglycerides were log transformed. All continuous variables were Z-standardized. T2D cases were excluded for all HOMA-IR analyses

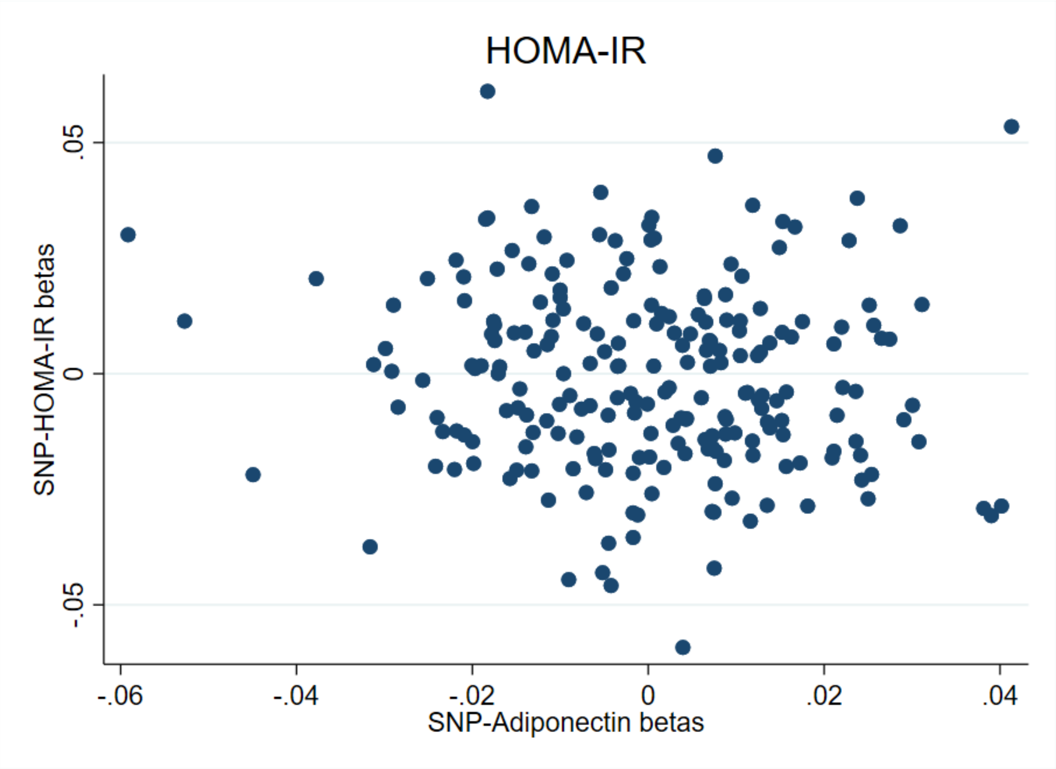
HOMA-IR
SNP-HOMA-IR betas
.05
0
-.05
-.06
-.04
-.02
0
.02
.04
SNP-Adiponectin betas

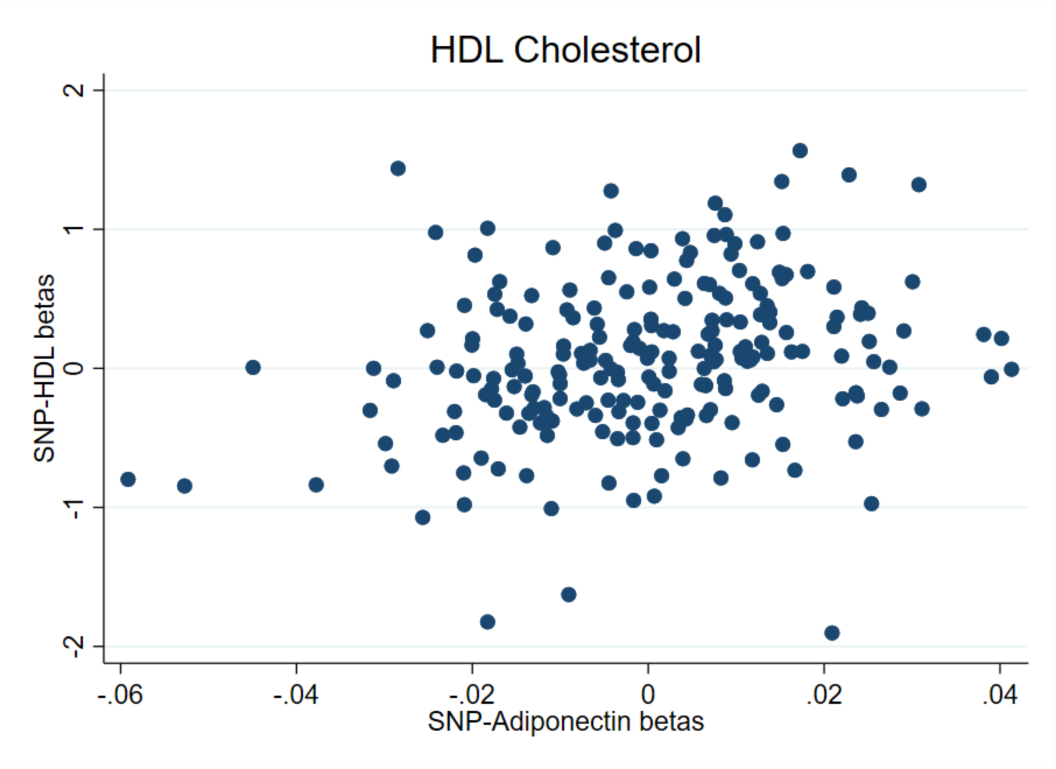
HDL Cholesterol
SNP-HDL betas
2
1
0
-1
-2
-.06
-.04
-.02
0
.02
.04
SNP-Adiponectin betas

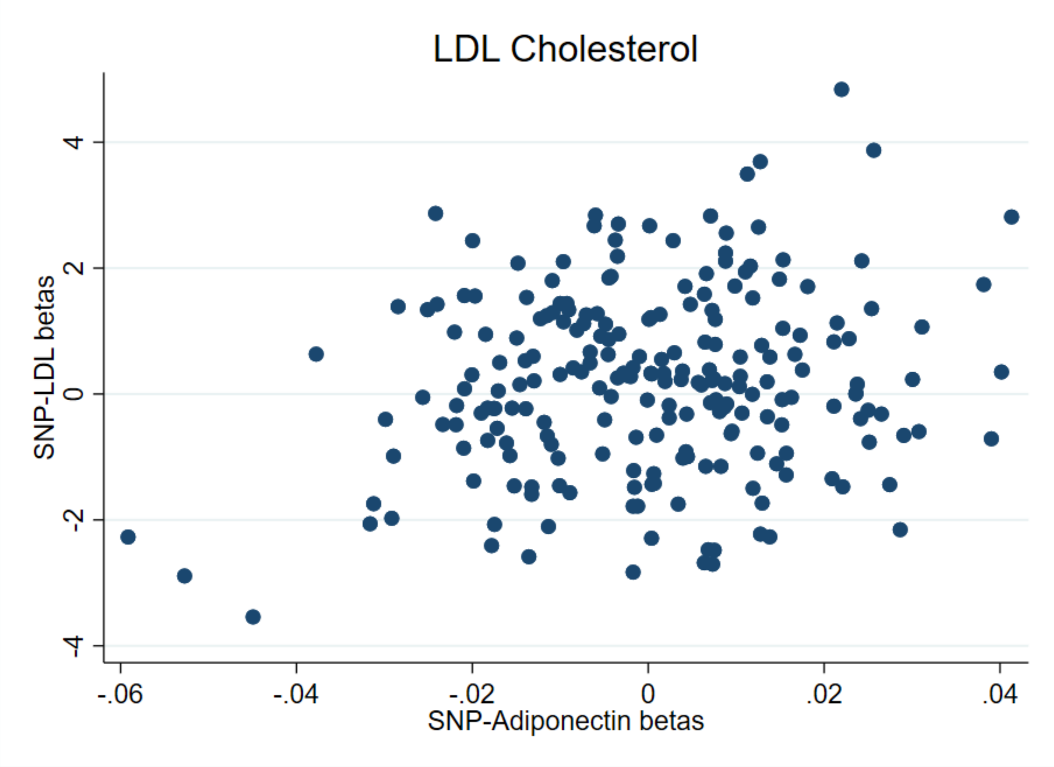
LDL Cholesterol
SNP-LDL betas
4
2
0
-2
-4
-.06
-.04
-.02
0
.02
.04
SNP-Adiponectin betas

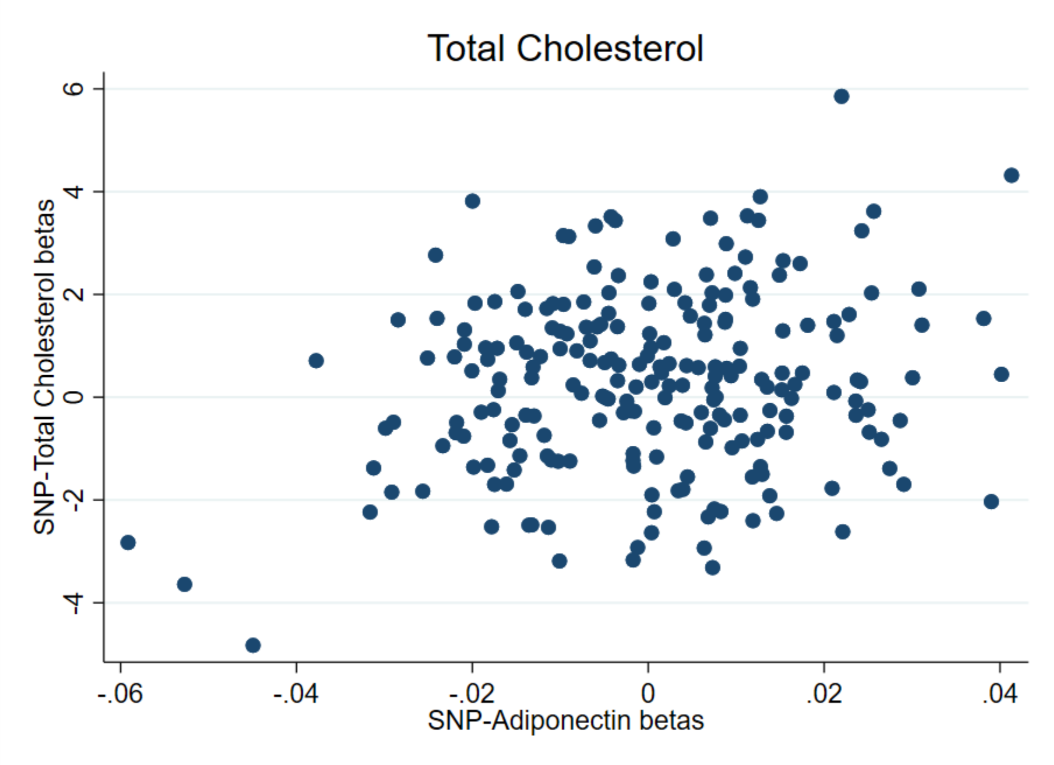
Total Cholesterol
SNP-Total Cholesterol betas
6
4
2
0
-2
-4
-.06
-.04
-.02
0
.02
.04
SNP-Adiponectin betas

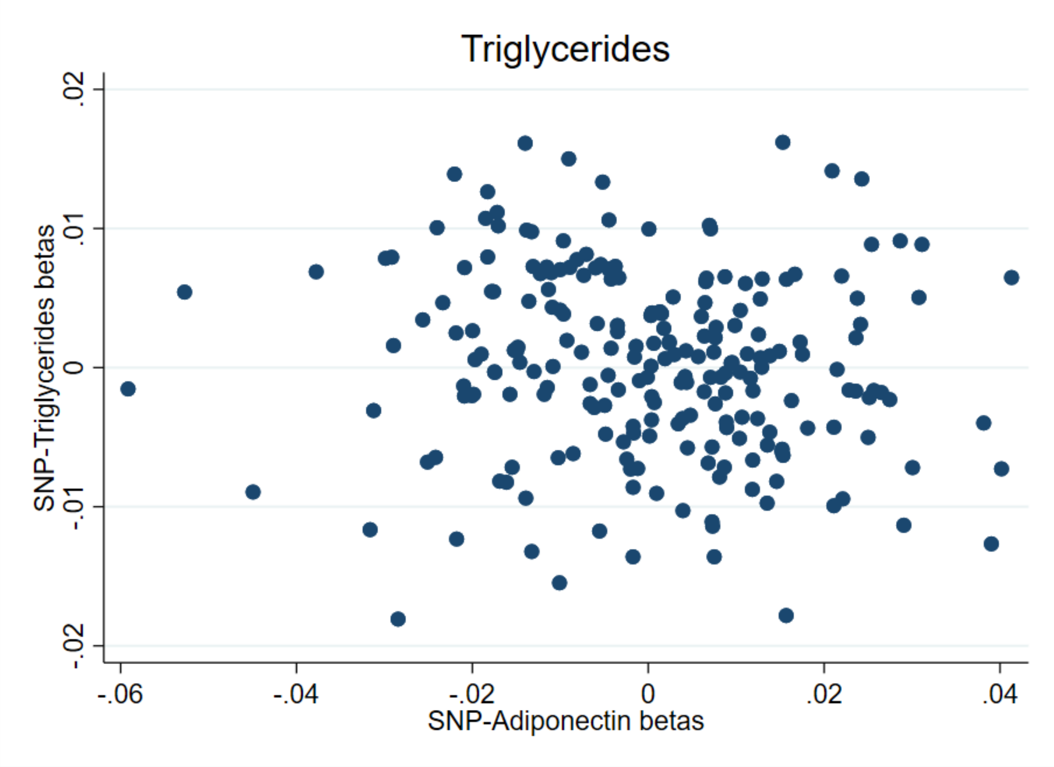
Triglycerides
SNP-Triglycerides betas
-.02
-.01
0
.01
.02
-.06
-.04
-.02
0
.02
.04
SNP-Adiponectin betas

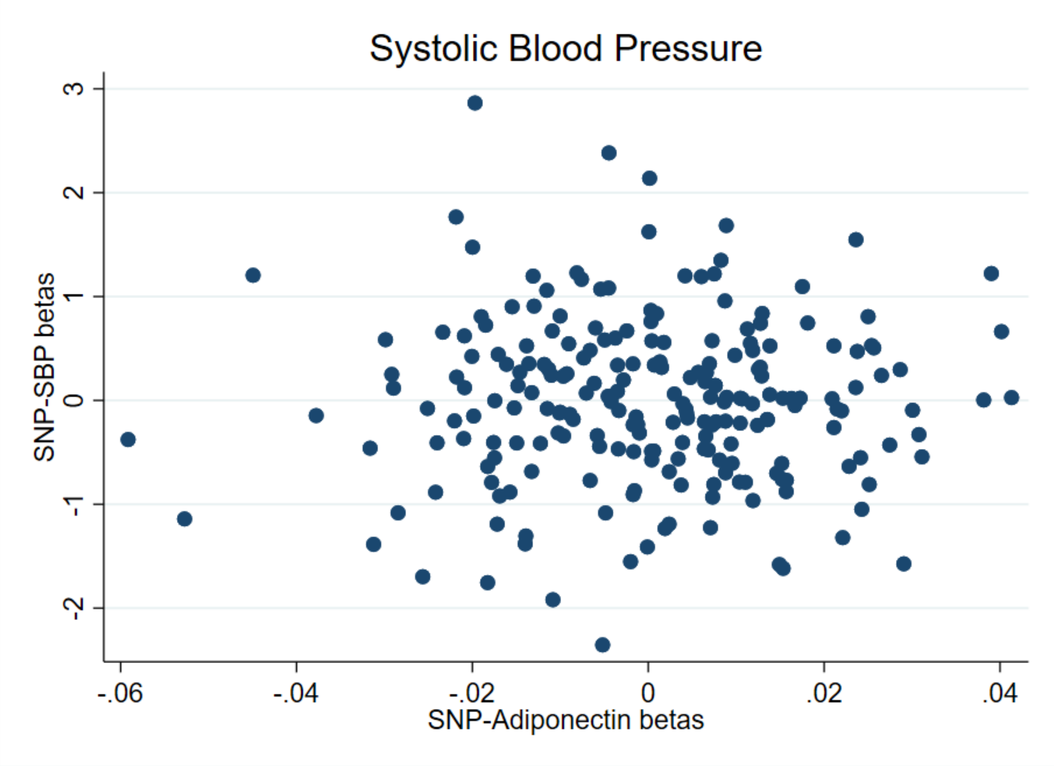
Systolic Blood Pressure
SNP-SBP betas
-2
-1
0
1
2
3
-.06
-.04
-.02
0
.02
.04
SNP-Adiponectin betas

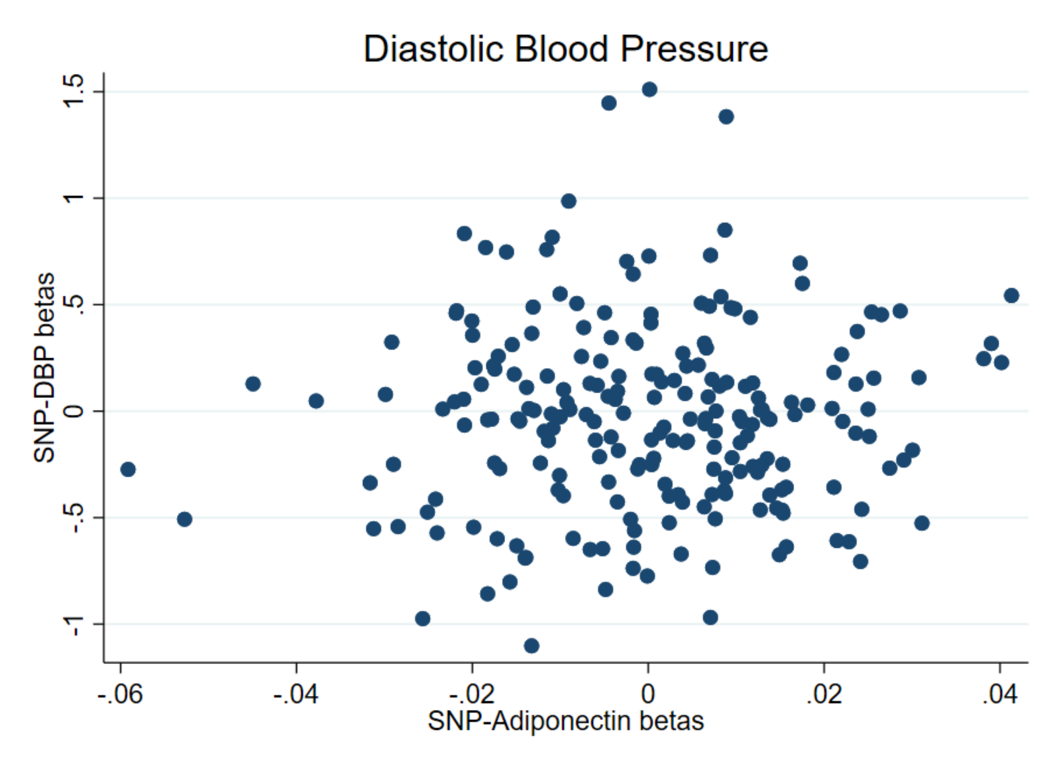
Diastolic Blood Pressure
SNP-DBP betas
1.5
1
.5
0
-.5
-1
-.06
-.04
-.02
0
.02
.04
SNP-Adiponectin betas

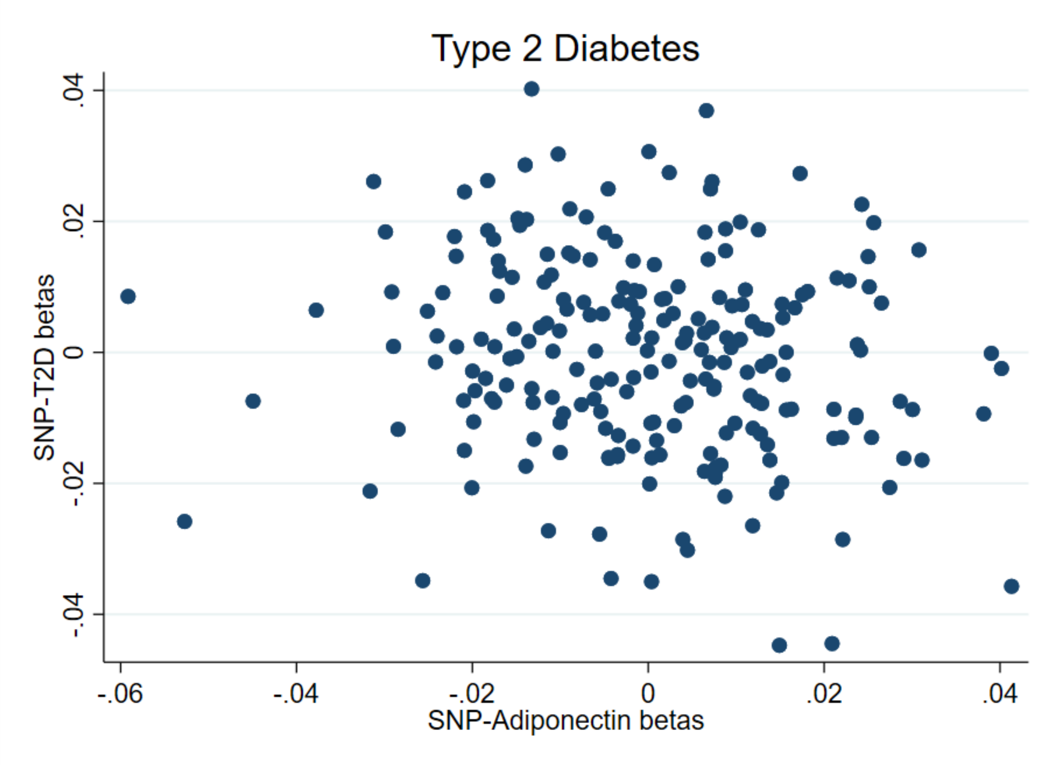
Type 2 Diabetes
SNP-T2D betas
.04
.02
0
-.02
-.04
-.06
-.04
-.02
0
.02
.04
SNP-Adiponectin betas

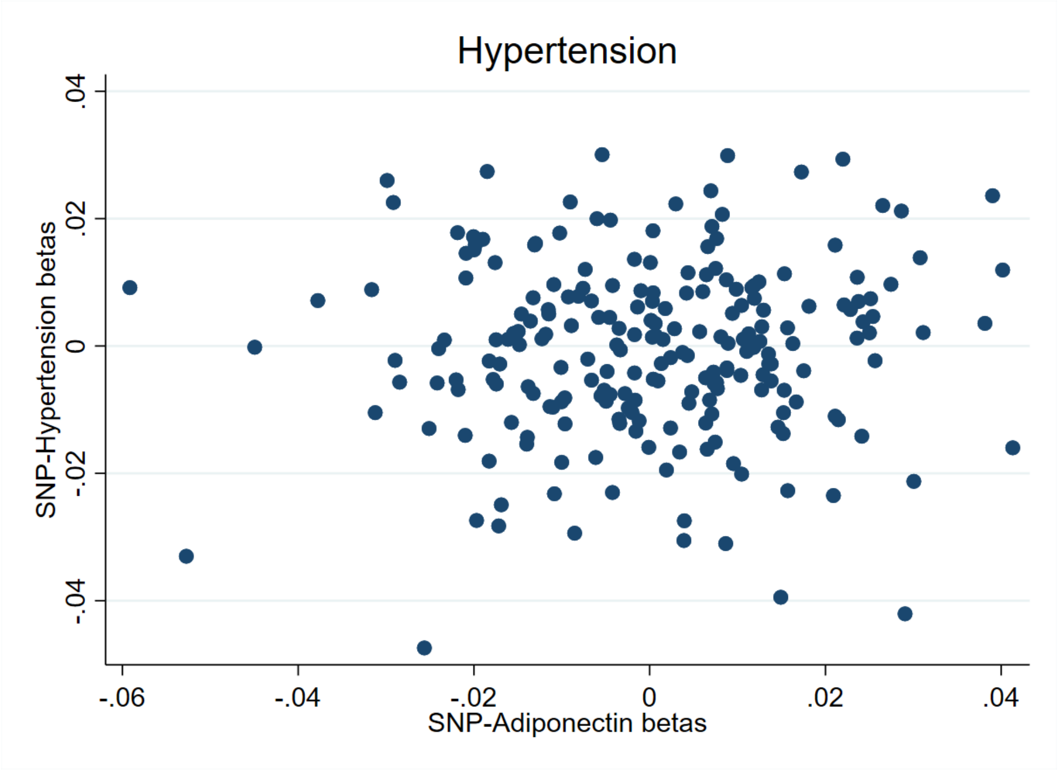

Figure S7. Scatter plots of SNP-adiponectin associations against SNP-outcome associations for the PRS SNPs.

Table S1. GWAS summary statistics for the adiponectin PRS comprising SNPs.

GWAS analyses were adjusted for age, sex, the first three principal components, and the genetic relationship matrix.

rsid	chr	position	ref allele	alt allele	beta	SE	P-value	Nearest gene
rs3128126	1	962210	A	G	-0.0042	0.0133	7.51E-01	*AGRN*
rs3766192	1	1017197	C	T	-0.0110	0.0151	4.66E-01	*C1orf159*
rs16850635	1	14961083	G	A	-0.0130	0.0124	2.96E-01	*KAZN*
rs2294888	1	14965569	G	A	-0.0024	0.0140	8.62E-01	*KAZN*
rs6424169	1	24590691	G	A	-0.0073	0.0128	5.66E-01	*AL590683.2*
rs10794657	1	24626174	A	G	0.0024	0.0116	8.37E-01	*RP11-10N16.3*
rs230271	1	40290597	C	A	-0.0140	0.0181	4.40E-01	*TRIT1*
rs12748241	1	159949360	G	A	0.0228	0.0168	1.73E-01	*LINC01133*
rs6680476	1	178676965	A	C	-0.0100	0.0137	4.65E-01	*RP11-428K3.1*
rs1531096	1	219656701	G	T	-0.0034	0.0121	7.78E-01	*RP11-95P13.1*
rs2066153	1	219663516	C	T	-0.0234	0.0127	6.64E-02	*RP11-95P13.1*
rs2820465	1	219675209	G	T	-0.0290	0.0119	1.53E-02	*RP11-95P13.2*
rs2785990	1	219687432	C	T	-0.0097	0.0196	6.23E-01	*RP11-95P13.2*
rs3001032	1	219727779	T	C	-0.0066	0.0115	5.66E-01	*RP11-95P13.2*
rs4637174	2	48960317	A	G	-0.0012	0.0144	9.34E-01	*LHCGR*
rs13020003	2	48976886	A	C	-0.0312	0.0137	2.31E-02	*LHCGR*
rs10198569	2	85794415	A	G	-0.0096	0.0126	4.44E-01	*VAMP8*
rs10460259	2	148704721	A	C	0.0107	0.0134	4.26E-01	*ORC4*
rs10184004	2	165508389	C	T	-0.0139	0.0143	3.31E-01	*COBLL1*
rs6705646	2	165567695	A	G	-0.0045	0.0195	8.17E-01	*COBLL1*
rs2943646	2	227099534	A	G	-0.0190	0.0122	1.19E-01	*AC068138.1*
rs2138157	2	227103717	A	C	-0.0292	0.0139	3.64E-02	*AC068138.1*
rs2943657	2	227123439	C	T	-0.0299	0.0135	2.70E-02	*AC068138.1*
rs2713549	2	227157209	A	G	-0.0150	0.0170	3.79E-01	*AC068138.1*
rs4663291	2	239023615	T	C	-0.0242	0.0138	8.00E-02	*ESPNL*
rs259553	3	21923119	A	G	-0.0218	0.0120	6.86E-02	*ZNF385D*
rs9867989	3	24291197	T	C	-0.0118	0.0120	3.26E-01	*THRB*
rs13074743	3	29986918	G	A	0.0301	0.0135	2.61E-02	*RBMS3*
rs7650825	3	35012263	A	G	-0.0061	0.0143	6.67E-01	*FECHP1*
rs4683297	3	46973673	T	C	0.0167	0.0148	2.59E-01	*CCDC12*
rs11130110	3	47000606	T	C	0.0135	0.0139	3.30E-01	*CCDC12*
rs13094438	3	47277885	G	A	0.0073	0.0137	5.97E-01	*KIF9*
rs1076394	3	47322781	G	A	0.0015	0.0146	9.17E-01	*KIF9*
rs3816779	3	47543389	G	A	0.0215	0.0143	1.33E-01	*ELP6*
rs614288	3	52220203	T	C	0.0060	0.0120	6.14E-01	*ALDOAP1*
rs648514	3	52467263	G	A	-0.0108	0.0130	4.06E-01	*SEMA3G*
rs12489828	3	52567014	G	T	0.0402	0.0120	8.04E-04	*NT5DC2*

rs1108842	3	52720080	A	C	0.0236	0.0118	4.45E-02	*GNL3*
rs3617	3	52833805	C	A	0.0390	0.0139	5.07E-03	*ITIH3*
rs9831977	3	52854140	T	G	0.0002	0.0253	9.95E-01	*ITIH4*
rs11130329	3	52896855	C	A	-0.0209	0.0141	1.37E-01	*TMEM110*
rs2581777	3	53054727	T	C	0.0153	0.0174	3.78E-01	*SFMBT1*
rs1346719	3	53077372	T	C	0.0146	0.0125	2.41E-01	*SFMBT1*
rs9841882	3	107909034	T	C	-0.0316	0.0206	1.25E-01	*IFT57*
rs9857471	3	113092978	T	G	-0.0093	0.0121	4.43E-01	*WDR52*
rs1567781	3	113294153	C	T	0.0129	0.0117	2.70E-01	*SIDT1*
rs9828868	3	126129646	C	T	-0.0070	0.0127	5.79E-01	*CCDC37*
rs11720677	3	126144746	C	T	0.0088	0.0178	6.21E-01	*CCDC37*
rs6765930	3	129020778	A	G	-0.0157	0.0220	4.75E-01	*HMCES*
rs4301033	3	150042618	G	A	-0.0146	0.0135	2.78E-01	*RP11-483E7.1*
rs1597466	3	150055561	G	T	0.0003	0.0120	9.80E-01	*RP11-483E7.1*
rs9862778	3	150116149	G	A	0.0039	0.0221	8.58E-01	*TSC22D2*
rs13081339	3	163543844	T	C	0.0019	0.0121	8.74E-01	*AC092962.1*
rs7649974	3	171409603	A	G	0.0034	0.0121	7.78E-01	*PLD1*
rs1828671	3	171581573	G	T	0.0070	0.0149	6.40E-01	*TMEM212*
rs9870756	3	186344614	C	T	-0.0042	0.0216	8.45E-01	*AHSG*
rs11918289	3	186431432	G	A	0.0048	0.0117	6.82E-01	*RP11-573D15.8*
rs1648722	3	186448989	C	T	0.0089	0.0120	4.56E-01	*KNG1*
rs3856930	3	186458322	C	T	0.0311	0.0202	1.24E-01	*KNG1*
rs710446	3	186459927	T	C	0.0045	0.0118	7.05E-01	*KNG1*
rs5030091	3	186460877	T	C	0.0024	0.0123	8.47E-01	*KNG1*
rs2062632	3	186461181	T	C	-0.0089	0.0199	6.55E-01	*KNG1*
rs1972703	3	186463343	G	A	-0.0251	0.0252	3.20E-01	*RP11-573D15.8*
rs822368	3	186466858	G	A	-0.0046	0.0116	6.95E-01	*RP11-573D15.8*
rs266743	3	186475791	T	C	0.0163	0.0130	2.11E-01	*RP11-573D15.8*
rs822354	3	186480206	A	G	-0.0209	0.0154	1.75E-01	*PSMD10P2*
rs843991	3	186516428	T	C	-0.0175	0.0118	1.39E-01	*RFC4*
rs16861184	3	186520477	C	T	0.0287	0.0216	1.84E-01	*RFC4*
rs6810075	3	186548565	T	C	-0.0136	0.0121	2.61E-01	*RP11-573D15.1*
rs10937273	3	186549695	G	A	0.0243	0.0180	1.77E-01	*RP11-573D15.1*
rs864265	3	186554292	T	G	-0.0592	0.0175	7.25E-04	*ADIPOQ*
rs16861209	3	186563114	C	A	0.0257	0.0154	9.68E-02	*ADIPOQ*
rs3774261	3	186571559	A	G	-0.0152	0.0119	2.01E-01	*ADIPOQ*
rs2117986	3	186600420	T	C	0.0181	0.0132	1.70E-01	*RPS20P14*
rs2412664	4	56457387	A	C	-0.0048	0.0173	7.79E-01	*PDCL2*
rs13434995	4	56467214	A	G	-0.0183	0.0181	3.14E-01	*NMU*
rs13133548	4	89740128	G	A	-0.0171	0.0118	1.47E-01	*FAM13A*
rs13140033	4	103363860	G	A	-0.0020	0.0147	8.91E-01	*RP11-499E18.1*
rs4241752	4	183721852	G	A	-0.0016	0.0125	9.00E-01	*TENM3*
rs1530962	5	8059876	C	T	0.0111	0.0189	5.58E-01	*RNU1-76P*
rs788547	5	29524508	C	T	0.0152	0.0132	2.48E-01	*UBL5P1*

rs10060696	5	39009131	A	G	-0.0017	0.0137	9.00E-01	*RICTOR*
rs4865796	5	53272664	G	A	-0.0017	0.0137	9.04E-01	*ARL15*
rs6450176	5	53298025	G	A	-0.0123	0.0128	3.36E-01	*ARL15*
rs13167840	5	104728701	T	C	0.0250	0.0140	7.37E-02	*RP11-6N13.1*
rs757537	5	132151071	A	G	-0.0017	0.0249	9.45E-01	*SOWAHA*
rs4705974	5	132198942	C	T	0.0077	0.0132	5.59E-01	*GDF9*
rs4704954	5	158172225	C	A	-0.0527	0.0239	2.75E-02	*EBF1*
rs10476266	5	158193818	C	T	-0.0240	0.0140	8.63E-02	*EBF1*
rs6914253	6	9824464	G	A	0.0124	0.0140	3.76E-01	*OFCC1*
rs855364	6	9834507	G	A	0.0291	0.0179	1.05E-01	*OFCC1*
rs1934772	6	9851995	G	A	-0.0185	0.0172	2.84E-01	*OFCC1*
rs969527	6	9986985	A	G	-0.0172	0.0124	1.66E-01	*OFCC1*
rs9477823	6	10096568	C	T	-0.0115	0.0126	3.62E-01	*OFCC1*
rs17606174	6	16687545	C	T	0.0308	0.0214	1.51E-01	*ATXN1*
rs3812200	6	16695530	C	T	0.0057	0.0120	6.36E-01	*ATXN1*
rs6912327	6	34764922	T	C	-0.0109	0.0139	4.31E-01	*UHRF1BP1*
rs998584	6	43757896	C	A	-0.0138	0.0168	4.11E-01	*VEGFA*
rs2065169	6	126191394	C	T	0.0087	0.0129	5.01E-01	*NCOA7*
rs3861397	6	139828916	A	G	-0.0183	0.0255	4.74E-01	*RP11-12A2.3*
rs592423	6	139840693	A	C	0.0104	0.0121	3.91E-01	*RP11-12A2.3*
rs596359	6	153457053	C	T	0.0136	0.0124	2.73E-01	*RGS17*
rs4709745	6	164105984	T	C	-0.0035	0.0127	7.83E-01	*RP1-230L10.1*
rs10242884	7	7154071	C	T	-0.0210	0.0128	1.00E-01	*Y_RNA*
rs320794	7	9734323	A	G	0.0039	0.0135	7.73E-01	*AC060834.2*
rs12669299	7	15739044	C	T	0.0073	0.0237	7.59E-01	*RPL36AP26*
rs1513275	7	28259233	T	C	-0.0115	0.0119	3.32E-01	*JAZF1-AS1*
rs7804534	7	129767636	G	A	-0.0378	0.0132	4.14E-03	*KLHDC10*
rs12706917	7	129779980	C	A	-0.0176	0.0150	2.42E-01	*RP11-775D22.2*
rs6971899	7	129788624	T	C	0.0013	0.0124	9.15E-01	*RP11-775D22.2*
rs7795852	7	140000505	G	A	0.0127	0.0153	4.04E-01	*SLC37A3*
rs1481692	8	13339368	G	A	-0.0028	0.0122	8.18E-01	*DLC1*
rs17116074	8	13348348	G	A	-0.0001	0.0129	9.94E-01	*DLC1*
rs4921994	8	18954802	C	T	0.0241	0.0138	8.06E-02	*RP11-*
rs2929564	8	73867626	A	G	0.0004	0.0166	9.82E-01	*RP11-531A24.3*
rs2980885	8	126474306	G	A	-0.0200	0.0174	2.52E-01	*RP11-136O12.2*
rs2980884	8	126474356	G	A	-0.0081	0.0172	6.37E-01	*RP11-136O12.2*
rs4871603	8	126480367	C	T	-0.0201	0.0117	8.76E-02	*RP11-136O12.2*
rs2980880	8	126480972	G	A	-0.0076	0.0122	5.35E-01	*RP11-136O12.2*
rs2954026	8	126484526	T	G	-0.0285	0.0244	2.44E-01	*RP11-136O12.2*
rs688784	9	13704356	A	G	-0.0058	0.0119	6.27E-01	*RP11-536O18.1*
rs524126	9	13708607	T	C	0.0004	0.0136	9.77E-01	*LINC00583*
rs12552748	9	26551100	T	C	0.0065	0.0145	6.55E-01	*AL442639.1*
rs2767755	9	126890847	G	A	0.0275	0.0130	3.41E-02	*None*
rs12341378	9	140837540	G	T	-0.0010	0.0129	9.38E-01	*CACNA1B*

rs10905791	10	5688085	C	T	-0.0133	0.0170	4.36E-01	*ASB13*
rs2782980	10	115781527	T	C	0.0004	0.0116	9.72E-01	*ADRB1*
rs7919873	10	115810668	A	C	0.0094	0.0127	4.60E-01	*ADRB1*
rs10787516	10	115813924	C	T	0.0209	0.0191	2.74E-01	*ADRB1*
rs1860404	10	119019177	T	C	0.0138	0.0127	2.77E-01	*SLC18A2*
rs11041308	11	7332512	T	C	0.0006	0.0119	9.58E-01	*SYT9*
rs1487853	11	7350851	C	T	0.0088	0.0119	4.58E-01	*SYT9*
rs11023139	11	14224346	G	A	0.0221	0.0181	2.22E-01	*SPON1*
rs1864658	11	14256444	T	C	-0.0090	0.0254	7.22E-01	*SPON1*
rs4757244	11	14258655	G	A	0.0065	0.0127	6.07E-01	*SPON1*
rs7941132	11	14302756	T	G	-0.0219	0.0151	1.48E-01	*RRAS2*
rs7938266	11	14709324	A	G	0.0220	0.0206	2.85E-01	*PDE3B*
rs12800057	11	65564987	G	T	-0.0033	0.0163	8.38E-01	*OVOL1*
rs887782	12	9084678	A	G	0.0042	0.0118	7.23E-01	*PHC1*
rs274982	12	20391365	A	G	0.0009	0.0126	9.40E-01	*CTC-465D4.1*
rs11045163	12	20463526	A	G	0.0081	0.0132	5.39E-01	*RP11-284H19.1*
rs7955516	12	20498036	A	C	0.0044	0.0133	7.43E-01	*RP11-284H19.1*
rs7965852	12	20511682	A	C	-0.0114	0.0125	3.63E-01	*RP11-284H19.1*
rs11045265	12	20614084	T	G	0.0071	0.0120	5.56E-01	*PDE3A*
rs2638319	12	56857057	C	T	0.0128	0.0150	3.95E-01	*MIP*
rs7302925	12	56861458	A	G	-0.0066	0.0123	5.92E-01	*MIP*
rs7314242	12	56876372	C	A	0.0116	0.0121	3.37E-01	*GLS2*
rs2657888	12	56938383	T	G	-0.0050	0.0121	6.83E-01	*RBMS2*
rs17250924	12	91061042	A	G	0.0153	0.0222	4.90E-01	*RP11-632B21.1*
rs10778510	12	107162965	G	A	-0.0131	0.0119	2.71E-01	*RP11-144F15.1*
rs4964494	12	107284460	A	G	-0.0178	0.0130	1.69E-01	*RIC8B*
rs2393326	12	118253345	C	T	-0.0161	0.0135	2.32E-01	*KSR2*
rs10850904	12	118255232	C	T	-0.0133	0.0177	4.53E-01	*KSR2*
rs1720033	12	122384344	A	G	0.0138	0.0151	3.59E-01	*WDR66*
rs7976716	12	122479987	T	C	0.0068	0.0172	6.92E-01	*BCL7A*
rs11043307	12	122494845	C	T	0.0071	0.0160	6.58E-01	*BCL7A*
rs9668430	12	122623595	A	G	0.0075	0.0144	6.05E-01	*MLXIP*
rs2017594	12	122631208	G	T	0.0238	0.0124	5.47E-02	*MLXIP*
rs925460	12	122634003	T	C	0.0265	0.0129	4.01E-02	*MLXIP*
rs10846688	12	122843225	G	A	0.0099	0.0120	4.13E-01	*CLIP1*
rs2454722	12	123171218	A	G	0.0018	0.0118	8.81E-01	*HCAR1*
rs10773036	12	124276909	C	T	0.0211	0.0115	6.70E-02	*DNAH10*
rs4930731	12	124317416	A	G	0.0073	0.0136	5.91E-01	*DNAH10*
rs7133378	12	124409502	G	A	0.0105	0.0116	3.68E-01	*DNAH10*
rs863750	12	124505444	C	T	-0.0155	0.0117	1.84E-01	*FAM101A*
rs1716403	12	124519846	T	C	-0.0102	0.0120	3.92E-01	*FAM101A*
rs12873640	13	85324940	T	C	0.0119	0.0144	4.08E-01	*LINC00333*
rs7985343	13	88134355	A	G	0.0130	0.0203	5.24E-01	*MIR4500HG*
rs9300546	13	100087719	T	G	0.0254	0.0229	2.67E-01	*AL583784.1*

rs9300812	13	104038402	G	A	0.0089	0.0139	5.22E-01	*RP11-123H22.1*
rs8016513	14	75238261	T	C	0.0157	0.0120	1.91E-01	*YLPM1*
rs3213717	14	75378347	C	T	-0.0100	0.0221	6.50E-01	*RPS6KL1*
rs8007184	14	100797892	C	T	0.0001	0.0162	9.96E-01	*SLC25A47*
rs8042229	15	32068300	G	A	0.0236	0.0146	1.06E-01	*OTUD7A*
rs4777839	15	93868240	C	A	0.0175	0.0130	1.77E-01	*RP11-266O8.1*
rs12598117	16	9118815	A	G	0.0083	0.0140	5.56E-01	*RP11-473I1.6*
rs4985155	16	15129459	A	G	-0.0220	0.0122	7.21E-02	*PDXDC1*
rs16948031	16	49863504	G	A	-0.0101	0.0123	4.12E-01	*ZNF423*
rs247611	16	56981240	G	A	0.0251	0.0124	4.23E-02	*HERPUD1*
rs10493891	16	81510742	C	T	0.0413	0.0224	6.57E-02	*CMIP*
rs12932649	16	81526836	C	T	0.0149	0.0193	4.39E-01	*CMIP*
rs2925979	16	81534790	T	C	0.0382	0.0126	2.40E-03	*CMIP*
rs2966093	16	81538620	G	A	-0.0175	0.0168	2.99E-01	*CMIP*
rs8191256	16	82133858	C	T	-0.0449	0.0251	7.33E-02	*RP11-510J16.5*
rs9889106	16	82481949	G	A	0.0043	0.0121	7.23E-01	*snoU13*
rs10514540	16	82482365	G	A	0.0075	0.0220	7.33E-01	*snoU13*
rs8058318	16	82628245	A	G	0.0064	0.0148	6.66E-01	*RP11-2L4.1*
rs3865181	16	82631672	A	G	-0.0017	0.0128	8.93E-01	*RP11-2L4.1*
rs8062637	16	82635368	T	C	0.0105	0.0128	4.15E-01	*CDH13*
rs3844410	16	82637637	T	G	0.0152	0.0122	2.11E-01	*CDH13*
rs7200895	16	82644606	C	T	0.0113	0.0135	4.04E-01	*CDH13*
rs4783244	16	82662268	G	T	-0.0060	0.0146	6.81E-01	*CDH13*
rs3852729	16	82668534	G	T	0.0037	0.0115	7.45E-01	*CDH13*
rs4782722	16	82672165	G	T	-0.0035	0.0159	8.26E-01	*CDH13*
rs12922394	16	82672327	C	T	-0.0055	0.0133	6.78E-01	*CDH13*
rs3910232	16	82673410	C	T	-0.0085	0.0150	5.68E-01	*CDH13*
rs1870843	16	82759314	G	A	0.0003	0.0121	9.79E-01	*CDH13*
rs1462046	16	82799127	T	C	0.0125	0.0159	4.30E-01	*CDH13*
rs12446293	16	82823465	T	C	0.0064	0.0157	6.84E-01	*CDH13*
rs6565065	16	82823552	A	G	0.0066	0.0122	5.87E-01	*CDH13*
rs8052008	16	82898799	G	A	-0.0045	0.0162	7.82E-01	*CDH13*
rs12929479	16	82997853	G	A	0.0157	0.0148	2.89E-01	*CDH13*
rs7203988	16	83042938	A	C	0.0007	0.0119	9.54E-01	*CDH13*
rs7500448	16	83045790	A	G	-0.0052	0.0249	8.35E-01	*CDH13*
rs8057717	16	83059913	A	C	0.0030	0.0136	8.28E-01	*CDH13*
rs4290460	16	83072462	C	A	-0.0199	0.0142	1.62E-01	*CDH13*
rs4558403	16	83663061	A	C	0.0076	0.0172	6.57E-01	*CDH13*
rs1437129	16	83705369	A	G	-0.0054	0.0118	6.46E-01	*CDH13*
rs12445022	16	87575332	G	A	0.0173	0.0191	3.66E-01	*RP11-482M8.1*
rs507506	17	7118322	A	G	-0.0037	0.0121	7.57E-01	*DLG4*
rs5415	17	7184481	T	C	-0.0197	0.0249	4.29E-01	*SLC2A4*
rs12051658	17	34315633	A	G	-0.0169	0.0195	3.87E-01	*CCL14*
rs3817293	17	78899595	G	A	0.0119	0.0120	3.23E-01	*RPTOR*

rs12955757	18	14095439	A	C	0.0118	0.0183	5.17E-01	*ZNF519*
rs11081575	18	77707525	G	T	0.0095	0.0138	4.91E-01	*PQLC1*
rs889140	19	33889000	G	A	0.0087	0.0135	5.19E-01	*PEPD*
rs3786897	19	33893008	A	G	0.0076	0.0117	5.15E-01	*PEPD*
rs731839	19	33899065	G	A	-0.0014	0.0120	9.09E-01	*PEPD*
rs4805885	19	33906123	T	C	0.0211	0.0117	7.01E-02	*PEPD*
rs7252630	19	39184822	T	G	-0.0148	0.0120	2.15E-01	*ACTN4*
rs927171	20	56232583	C	A	-0.0256	0.0211	2.24E-01	*PMEPA1*
rs2823158	21	16591544	A	G	0.0028	0.0117	8.08E-01	*AJ006998.2*
